# A Multilayer Network Approach for Guiding Drug Repositioning in Neglected Diseases

**DOI:** 10.1371/journal.pntd.0004300

**Published:** 2016-01-06

**Authors:** Ariel José Berenstein, María Paula Magariños, Ariel Chernomoretz, Fernán Agüero

**Affiliations:** 1 Laboratorio de Bioinformática, Fundación Instituto Leloir, Buenos Aires, Argentina; 2 Departamento de Física, Universidad de Buenos Aires, Buenos Aires, Argentina; 3 Laboratorio de Genómica y Bioinformática, Instituto de Investigaciones Biotecnológicas–Instituto Tecnológico de Chascomús, Universidad de San Martín–CONICET, Sede San Martín, San Martín, Buenos Aires, Argentina; McGill University, CANADA

## Abstract

Drug development for neglected diseases has been historically hampered due to lack of market incentives. The advent of public domain resources containing chemical information from high throughput screenings is changing the landscape of drug discovery for these diseases. In this work we took advantage of data from extensively studied organisms like human, mouse, *E*. *coli* and yeast, among others, to develop a novel integrative network model to prioritize and identify candidate drug targets in neglected pathogen proteomes, and bioactive drug-like molecules. We modeled genomic (proteins) and chemical (bioactive compounds) data as a multilayer weighted network graph that takes advantage of bioactivity data across 221 species, chemical similarities between 1.7 10^5^ compounds and several functional relations among 1.67 10^5^ proteins. These relations comprised orthology, sharing of protein domains, and shared participation in defined biochemical pathways. We showcase the application of this network graph to the problem of prioritization of new candidate targets, based on the information available in the graph for known compound-target associations. We validated this strategy by performing a cross validation procedure for known mouse and *Trypanosoma cruzi* targets and showed that our approach outperforms classic alignment-based approaches. Moreover, our model provides additional flexibility as two different network definitions could be considered, finding in both cases qualitatively different but sensible candidate targets. We also showcase the application of the network to suggest targets for orphan compounds that are active against *Plasmodium falciparum* in high-throughput screens. In this case our approach provided a reduced prioritization list of target proteins for the query molecules and showed the ability to propose new testable hypotheses for each compound. Moreover, we found that some predictions highlighted by our network model were supported by independent experimental validations as found *post-facto* in the literature.

## Author Summary

Neglected tropical diseases are human infectious diseases that are often associated with poverty. Historically, lack of interest from the pharmaceutical industry resulted in the lack of good drugs to combat the majority of the pathogens that cause these diseases. Recently, the availability of open chemical information has increased with the advent of public domain chemical resources and the release of data from high throughput screening assays. Our aim in this work was to make use of data from extensively studied organisms like human, mouse, *E*. *coli* and yeast, among others, to prioritize and identify candidate drug targets in neglected pathogen proteomes, and drug-like bioactive molecules to foster drug development against neglected diseases. Our approach to the problem relied on applying bioinformatics and computational biology strategies to model large datasets spanning complete proteomes and extensive chemical information from publicly available sources. As a result, we were able to prioritize drug targets and identify potential targets for orphan bioactive drugs.

## Introduction

Neglected tropical diseases (NTDs) devastate the lives of approximately 1 billion people, with a further 1 billion at risk [1–3]. These diseases mainly affect those who live in poverty in Africa, Asia and the Americas. Current treatments for these diseases present several issues and limitations such as cost, difficulties in administration, poor safety profiles, lack of efficacy, and increasing drug resistance, among others [4]. Furthermore, there has been limited commercial interest in developing improved therapeutics, mostly because of the costly and risky nature of the drug discovery process [5,6] and the expected low return of investment when dealing with poor patient populations [7]. As a consequence, only ~1% of all new drugs that reached the market in recent years were for neglected diseases [1,4].

The situation for human diseases that affect the developed world is radically different. In this case, many important contributions to drug discovery are made every year from academic and government laboratories, leading to the approval of ~20 new drugs per year on average [8]. As part of this process of drug discovery, we accumulate information about many bioactive compounds (their activities, targets and mechanisms of action), which can be used in repositioning strategies.

Drug repositioning (or repurposing, or reprofiling) is the process of finding new indications for existing drugs [9]. The benefits of this approach are many, the main being the lower costs of development [5,9–11]. A number of success stories help support the case for these type of approaches. Two of the best known examples are sildenafil (Viagra), which was repositioned from a common hypertension drug to a therapy for erectile dysfunction [11] and thalidomide, repurposed to treat multiple myeloma and leprosy complications [12]. Because of the enormous cost savings associated with repositioning an approved drug, this strategy is particularly attractive for NTDs. For these, there are also a number of successful repositioning stories: eflornithine, which was developed as an anticancer compound is being used to treat African trypanosomiasis (sleeping sickness), whereas pentamidine, amphothericin B (originally an antifungal drug) and miltefosine were all repositioned from other indications for the chemotherapy of leishmaniasis (other examples were discussed recently, see [13,14]).

Target prioritization, and drug repositioning are particularly amenable to the use of computational data mining techniques, which offer high-level integration of available knowledge [15]. These strategies take advantage of bio- and chemoinformatic tools to make full use of known targets, drugs, and disease biomarkers or pathways, which in turn lead to a faster computer-to-bench or computer-to-clinic studies. Exploring a large pharmacological space in this way has led to novel insights on the targets and modes of action of existing drugs [16–24]. Unfortunately, these and other integrative mining strategies were focused in attacking the problem from the point of view of diseases of the developed world. Fortunately it is relatively straightforward to use a number of inference strategies to map informative associations to other species. Kruger and coworkers recently showed that ligand binding to > 150 human proteins is mostly conserved across mammalian orthologs, therefore providing support for this type of inferences [25].

It is also worthwhile mentioning that particularly in the case of neglected diseases, drug repositioning need not be taken in a strict sense to include only drugs approved for clinical use in humans. Widening the criteria to reposition drugs for veterinary use, or further, any bioactive compound (hits/leads) may significantly increase the chances of success by helping to guide efforts in academia and pharma. These will ultimately feed the pipeline of drug discovery for these important diseases.

After completion of a number of key pathogen genome projects, we developed a database resource to help prioritize candidate targets for drug discovery in NTDs [26,27]. Initially, target prioritizations were based on gene and protein features, with limited use of information on availability of bioactive compounds to guide these prioritizations. Since then we have integrated information on a large number of bioactive compounds into the TDRtargets.org database [28]. These were derived from public domain resources, and from a number of high-throughput screenings of an unusual scale for NTDs [29–31]. This has brought the current status of chemogenomics data integration in NTDs to a stage where large scale data mining exercises are now feasible.

Complex networks can efficiently describe pairwise similarity relations between drugs and between proteins. Under this paradigm non-trivial interconnectivity patterns can be mined to uncover hidden organization principles, or to identify unnoticed relevant entities and/or novel putative drug-target associations [18,23,32–40]. In this work we addressed the construction of a multilayer network of protein targets (gene products), chemical compounds, and their relations, in order to guide drug discovery efforts. Because we focused on tropical diseases, we were interested in leveraging the information contained in the network (mostly derived from well-studied organisms) to direct the selection of targets and compounds for further experimentation in these neglected pathogens. In this context we tackled two well differentiated problems. First, we analyzed the prioritization of targets for drug discovery in the absence or scarcity of bioactivity data for an organism of interest. For a selected pathogen (a query species), we took advantage of chemogenomics and bioactivity data available in the network, to get a global prioritized list of promising targets. In a second analysis, we used the information embedded in the network to suggest candidate targets for orphan compounds, i.e. chemicals that have been shown to be active in whole-cell or whole-organism screenings but whose targets are currently unknown. In this case, we aimed to obtain reduced prioritization lists of target proteins for the query molecule.

## Methods

### Data sources

All target data used in this work was obtained from the TDR Targets database [26,28], which includes complete genomes from a number of pathogens causing neglected tropical diseases, as well as model organisms: *Plasmodium falciparum*, *Trypanosoma brucei*, *Trypanosoma cruzi*, *Leishmania major*, *Mycobacterium tuberculosis*, *Brugia malayi*, *Schistosoma mansoni*, *Toxoplasma gondii*, *Plasmodium vivax*, *Leishmania braziliensis*, *Leishmania infantum*, *Leishmania mexicana*. In addition we integrated data from complete genomes from non-pathogen organisms: vertebrates (human, mouse), plantae (*Arabidopsis thaliana*, *Oryza sativa*), invertebrates (*Drosophila melanogaster*), and nematodes (*Caenorhabditis elegans*), fungi (*Saccharomyces cerevisiae*), and bacteria (*Escherichia coli*). Pfam domain annotations for all targets were obtained from the InterPro database resource, using interproscan [41]. Metabolic pathway, and EC number annotations for all targets were obtained from the KEGG database resource [42]. Orthology relationships between targets were obtained from the OrthoMCL database [43] or calculated by mapping proteins against OrthoMCL ortholog groups using BLASTP [44]. As a result we had our proteins mapped to 69,926 ortholog groups (a singleton is considered also as a separate ortholog group of size = 1). Information on chemical compounds (structures, bioactivity information) was obtained from the ChEMBL database [45]. This information was complemented by manually curated data from the TDR Targets database on compounds active against pathogens (see below).

### Defining relationships between chemical compounds

We estimated chemical similarity between molecules by performing an all vs all fingerprint-based similarity analysis using checkmol [46]. The algorithm for fingerprint generation has been described [46], but briefly, for each molecule the molecular graph is disassembled into all possible linear fragments with a length ranging from 3 to 8 atoms. Strings representing atom types as well as bond types of these linear fragments are then passed to two independent hash functions in order to compute two pseudo-random numbers in the range 1–512, which are used to set two positions in the 512-bit binary fingerprint. For similarity search operations, the hash-based fingerprint of the query structure was used to compute the Tanimoto similarity coefficient (Tc) [47] for each pairwise combination of query/candidate hash-based fingerprints. Because pairs of molecules with low Tc values have insubstantial chemical similarity, for the Drug-network layer we only considered similarity relationships with Tc values ≥0.8 as these are expected to be both significant in statistical terms [48] and in terms of their expected biological activity [49]. As a result we retained about 44.4 10^6^ informative pairwise relations and used the corresponding Tc values to weight the corresponding links.

In addition, for each bioactive molecule *d* ∈ *V*_*D*_, we identified exact substructure relationships using matchmol. These substructure relationships, unlike other similarity measurements, were asymmetrical (a 2D/graph representation of a molecule was completely included within another one, but not *viceversa*). We filtered out substructure relationships for very small molecules as these were more likely to be contained within larger and more complex molecules rather unspecifically without a strict correlation with expected targets or modes of action. After analyzing the distribution of molecular weight and number of parental structures of each compound (parental molecules are those that contain a compound as part of its structure) we filtered out edges involving molecules with low molecular weight (MW < 150) and large number of parental structures (*Nparents*>100). We found that the adopted molecular weight threshold appeared as a reasonable and conservative maximal bound for filtering out highly promiscuous structures (i.e. molecules included in more than 100 parental compounds). For larger molecular weights the number of affected molecules would have been much more sensitive to the adopted threshold level (see S3 Fig).

Taking into account Tanimoto similarities and substructure relationships, we set up the drug layer graph *G*_*D*_(*V*_*D*_ = {*d*_1_,…,*d*_*M*_}, *E* = {*c*_*ij*_}_*i*,*j* = 1…*M*_). We considered weighted inter-compounds edges *c*_*ij*_ ∈ *R*^(0+)^ defined as:

$$c_{ij}=\text{max}\left\{T_{C}\left(d_{i},d_{j}\right)*\;I\left(T_{C}\left(d_{i},d_{j}\right)\geq 0.80\right)\;,0.8*I\left(d_{i}\subset d_{j}\right)\right\}$$ (1)

where *I(x)* is an index function that equals 1 if its argument is a true proposition and 0 otherwise, and *d*_*i*_ ⊂ *d*_*j*_ means that *d*_*i*_ is an exact substructure of *d*_*j*_. In words, each substructure edge received a weight value of 0.8, and each valid Tanimoto edge (*T*_*c*_ ≥ 0.8) was weighted considering the corresponding *T*_*c*_ value. The overall chemical similarity information between a pair of compounds was then integrated into a single link taking into account the maximal available weight that could be established between them.

### Defining relationships between protein targets and chemical compounds

Links between compounds and proteins were derived from bioactivity information, obtained from different sources (ChEMBL, PubChem, TDR Targets), as well as a focused manual curation of the literature performed for this work. Due to the great diversity of assays and forms of reporting bioactivity values, we selected a number of assays for which we have the greatest amount of data, and we defined a cutoff value for each bioactivity type, in order to classify the compound as active or inactive (Table 1). The bioactivity classes that were taken into account represent 95% of the total bioactivities in our dataset. In the case of orphan compounds that are active against *P*. *falciparum* (see Results) bioactive molecules correspond to the assays detailed in the Table 2.

**Table 1:** Bioactivity types and activity cutoffs. The table lists the TDR Targets bioactivity types considered (as reported by paper authors or as imported by curators of the corresponding sources, the number of targets, compounds, and assays (bioactivity data points) in each case. The last two columns show the number of assays in which molecules were classified as active, and the corresponding cutoff value used in this classification. Pf = Plasmodium falciparum; DHOD = Dihydroorotate dehydrogenase; FP-2 = falcipain-2.

Dataset / Bioactivity	Compounds	Targets	Assays	Active	Cutoff
**Homozygous knockout**	95	3542	889,407	65,148	P value < 0.01
**Heterozygous knockout**	247	5857	3,572,775	154,535	P value < 0.01
**Various bioactivities**	142	24	397	148	< = 2 μM; > = 80%
**Pf DHOD EC50**	172	1	172	2	< = 2 μM
**Pf FP-2 EC50**	172	1	172	0	< = 2 μM
**I50**	2,240	97	3,502	1,145	< = 2 μM
**IC50**	152,722	2,238	297,136	184,866	< = 2 μM
**Inhibition**	29,604	1,404	55,659	9,350	> = 80%
**Kd**	3,034	440	5,697	3,923	< = 2 μM
**Activity**	5,898	654	12,804	3,751	> = 80%
**Ki**	77,368	1,519	181,578	134,904	< = 2 μM
**EC50**	16,221	528	30,089	20,961	< = 2 μM
**ED50**	1,550	117	2,361	1,240	< = 2 μM
**Efficacy**	2,748	102	5,346	1,900	> = 80%

**Table 2:** Bioactivity types derived from high-throughput screenings against *Plasmodium falciparum*. Sources are GSK TCAMS: GlaxoSmithKline Tres Cantos Antimalarial Screening [29]; Novartis-GNF: Novartis-GNF Malaria Box dataset [50]; and SJCRH: Saint Jude Children's Research Hospital [30].

Bioactivity type / Assay	Compounds	Bioactivities	Positive	Cutoff	Source
**% growth inhibition Pf 3D7 at 2 μM**	**13,469**	**13,519**	**13,484**	**> = 80%**	**GSK TCAMS**
**% growth inhibition Pf Dd2 at 2 μM**	13,469	13,519	5,061	> = 80%	GSK TCAMS
**EC50 Pf 3D7**	**5,387**	**5,497**	**4,523**	**2 μM**	**Novartis-GNF**
**EC50 Pf W2**	5,375	5,485	4,804	2 μM	Novartis-GNF
**EC50 Pf 3D7**	**172**	**172**	**152**	**2 μM**	**SJCRH**
**EC50 Pf V1/S**	172	172	141	2 μM	SJCRH
**EC50 Pf 3D7, SYBR green**	**1,524**	**1,536**	**496**	**2 μM**	**SJCRH**
**% growth inhibition at 7 μM**	1,524	3,072	2,475	> = 80%	SJCRH
**EC50 PfK1, by SYBR green**	**1,524**	**1,536**	**488**	**2 μM**	**SJCRH**
**EC50 PfD10_yDHOD**	172	172	136	2 μM	SJCRH
**EC50 PfDd2**	**172**	**172**	**158**	**2 μM**	**SJCRH**
**EC50 PfK1**	172	172	153	2 μM	SJCRH
**EC50 PfSB-A6**	**172**	**172**	**129**	**2 μM**	**SJCRH**
**EC50 PfW2**	172	172	116	2 μM	SJCRH

### Relevance score of affiliation-type nodes

For the i-th affiliation-type node, *f*_*i*_ ∈ *V*_*F*_ (which represents a shared functional relation among proteins, such as an ortholog group, a Pfam domain, or a defined biochemical pathway, we defined a *Relevance Score*, *RS*_*i*_, as a proxy of its informative relevance with regard to drug-target predictions tasks. To this end, we performed an overrepresentation test (Fisher exact test) to quantify the overrepresentation in each affiliation category of druggable proteins, where the criteria for druggability are the cutoffs described in Table 1. Taking into account the corresponding Fisher test p-value, *p*_*v*_^*i*^, we defined the attribute node’s r*elevance score* as

$$RS_{i}=-log_{10}(p_{v}{}^{i})$$ (2)

### Bipartite network projection and prioritization algorithms

The protein and affiliation node layers defined a bipartite graph which can be represented by an adjacency matrix $M^{bip}\in R^{n_{p}\times n_{f}}$:

$$M_{ij}^{bip}=\{\begin{array}{l} 1\;\text{if protein}\;i\;\text{is annotated to category}\;f_{j}\\ \;0\;\text{otherwise} \end{array}$$ (3)

We projected this bipartite network into a mono-partite graph, the Projected Protein Layer (*PP-layer)*, where protein nodes were connected through weighted links if they share common affiliation nodes. The corresponding adjacency matrix $M^{PP}\in R^{n_{p}\times n_{p}}$ was defined as

$$M^{PP}=M^{bip}\;S\;{\left(M^{bip}\right)}^{T}$$ (4)

where $S\in R^{n_{f}\times n_{f}}$ was a diagonal scoring matrix for affiliation nodes. We considered two alternative definitions for the scoring matrix *S*. In the first case, *S* = *S*^*r*^, diagonal elements were defined as

$$S^{r}{}_{ii}=f\left(RS_{i}\right)=\{\begin{array}{l} 1\;\text{if}\;RS_{i}\geq quantile(RS\;,0.8)\\ \;{\left(\frac{RS_{i}}{\text{max}\;\{RS_{i}\}}\right)}^{\alpha }\;\text{otherwise} \end{array}$$ (5)

where *α* was a tunable parameter that was set by maximizing the performance of recovering known druggable targets in cross validation exercises (see below)

For the second alternative, in view of the broad degree distribution observed for affiliation nodes, we also considered an extra factor that relativized the score of large categories. In this case diagonal elements of *S* = *S*^*r*^ were defined as

$$S^{rk}{}_{ii}=f\left(RS_{i}\right)=\{\begin{array}{l} \;\frac{1}{k_{i}}\;\text{if}\;RS_{i}\geq quantile(RS\;,0.8)\\ \frac{1}{k_{i}}{\left(\frac{RS_{i}}{\text{max}\;\{RS_{i}\}}\right)}^{\alpha }\;\text{otherwise} \end{array}$$ (6)

where *k*_*i*_ is the degree of the *i-th* affiliation node, and *α* was a tunable parameter (see below).

Both scoring matrices, *S*^*r*^ and *S*^*rk*^, led to different projected *PP-layers* and induced two alternative two-layered weighted graphs *G*'(*V* = {*V*_*D*_, *V*_*P*_}, *E* = {*E*_*DD*_, *E*_*DP*_, *E*_*PP*_}), namely $G_{r}^{'}$ and $G_{rk}^{'}$. These graphs were used to address different prioritization tasks throughout this manuscript. In either case the free parameter α was set by maximizing the performance of recovering druggable targets.

### Voting scheme prioritization

Let’s consider a weighted graph *G* = *G*(*V* = {*n*_*i*_}_*i* = 1…*N*`_, *E* = {*e*_*ij*_}_*i*,*j* = 1…*N*_), where $e_{ij}\in R^{0+}$ are weighted edges, and a vertex seed set *S* = {*s*_1_,…,*s*_*k*_}. The voting scheme assigns to each node *n*_*i*_ not included in the seed set a prioritization score, *PS*, according to the following expression:

$$PS_{i}=\underset{j=1\ldots k}{\sum }w_{j}e_{ji}$$ (7)

where *w*_*j*_ is a real number that serves to weight the contribution of seed *s*_*j*_, and *e*_*ji*_ the weight value of the link joining nodes *n*_*j*_ and *n*_*i*_. When we prioritized targets from a query proteome ***Q***, we set *w*_*j*_ = 1∀*j* (i.e. we considered uniform and equally weighted seeds). On the other hand, when we prioritized candidate targets for an orphan compound *d*_*k*_, we set *w*_*j*_ according to the similarity between *d*_*k*,_ and its direct neighbor drugs which reported bioactivities against protein *s*_*j*_:

$$w_{j}=\sum_{i:d_{i}\in N(d_{k})}c_{ki}e_{ij}^{DP}$$ (8)

where *c*_*ki*_ is the weight of the edge between *d*_*k*_ and *d*_*i*_ molecules introduced in Eq [1], *e*_*ij*_^*DP*^ is 1 if there was a bioactivity link between drug *d*_*i*_ and protein *p*_*j*_ (and *0* otherwise) and *N*(*d*_*k*_) the set of direct neighbors of drug *d*_*k*_.

### Parameter settings

The *PP-layer* results from a projection of a bipartite network graph. The procedure used for this projection is dependent on the single parameter α (see Eqs 2 and 3). In order to analyze the effect of α on the ability to recover known targets from an entire genome, we calculated ROC curves, and compared the partial AUC-0.1 for different α values following a tenfold cross validation procedure. The results are summarized in S4 Fig It can be noticed that the predictive performance remained near maximal, without significant variations, for a broad range of the parameter space, *α* ∈ [0.2, 1], suggesting that the method is robust to different α selections. From this point forward, we considered *α* = 0.6, the midpoint in this interval. An important remark is that *α* = 0 - which corresponds to disregarding the relevance score in the definition of the S matrix (see Eqs 4 and 5)—had a significantly lower performance than the *α* = 0.6 case (*p*_*v*_ < 10^−24^, Wilcoxon test).

## Results

### Multilayer network construction

We integrated genomic, biochemical and medicinal chemistry data from several public domain resources (see Methods). These data is available from the TDR Targets database and includes genome data from pathogen and model organisms. As a starting point we considered sequence information from ~ 1.7 10^5^ proteins derived from 37 complete genomes (S1 Table) and from known druggable targets from other 184 species. We also considered a number of affiliation-type features for these proteins, which would allow us to establish relations between proteins, like sharing of protein domains, clustering in the same ortholog groups and participation in the same metabolic pathways. These features were selected because they provide complementary information on the similarity of these proteins, from the point of view of drug discovery, and because they can be easily computed for whole genomes. In addition, we considered structural information from ~1.5 10^6^ bioactive compounds, and their associated bioactivity data against pathogen and non-pathogen organisms, obtained from open chemical databases and high throughput screenings [29–31,45,51].

**Fig 1:**
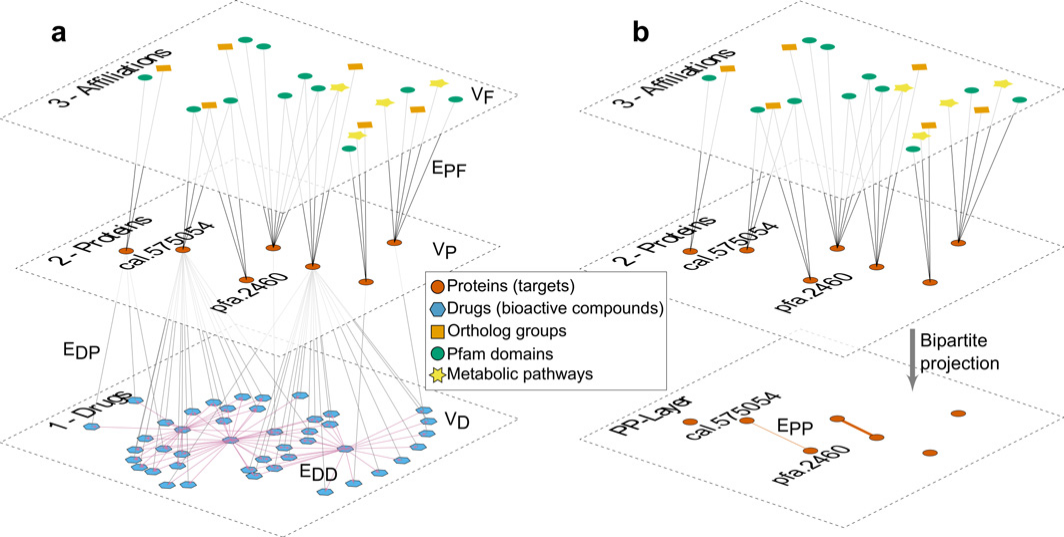
Schematic representation of data and workflow. **a)** Multilayer representation of drug-target data. First layer (bottom) contains drugs as nodes and chemical similarity relations as edges. Second layer contains proteins as nodes. Links between these two layers represent known and significant bioactivity of a compound against a defined protein target. The top layer contains functional annotation-type objects as nodes (Pfam domains, green circles; Ortholog groups, orange diamonds; and metabolic pathways, yellow stars). Links between the second and third layers represent affiliations of proteins to each of these annotation classes. For clarituy, the representation shows a partial view of the whole network corresponding to objects and connections related to the example shown in Fig 5. **b)** Bipartite projection of the two upper layers, into a protein-protein monopartite network after weighting of informative affiliations as described in the main text.

In order to organize and provide a global description of the available heterogeneous data, we considered a multipartite, multilayered network graph *G*(*V* = {*V*_*D*_,*V*_*P*_,*V*_*F*_}, *E* = {*E*_*DD*_, *E*_*DP*_, *E*_*PF*_}). In this network three types of vertices *V*_*D*_,*V*_*P*_,*V*_*F*_ represented bioactive compounds, proteins, and functional affiliation entities, respectively. Relationships between pairs of compounds, between compounds and known protein targets, and between proteins and functional affiliation classes where represented by the corresponding edges *E*_*DD*_, *E*_*DP*_, *E*_*PF*_. Fig 1A depicts a graphical representation of this network, where three layers, each including a different type of vertex can be recognized.

The first layer contained chemical compounds as nodes (*V*_*D*_ *= {d*_*1*_,*d*_*2*_,*…}*). Weighted pairwise links between compounds (E_DD_) were established if they were chemically similar based on their 2D representations. More specifically, we connected two compounds if the Tanimoto similarity coefficient of their 2D fingerprints was >0.8 (which is a very conservative similarity cutoff [48]), or if a compound was an exact substructure of the other. In this case the directionality of the relationship was preserved (see Methods for details).

Nodes in the second layer (*V*_*P*_ *= {p*_*1*_,*p*_*2*_,*…}*) represented proteins from 221 pathogen and non-pathogen (model) organisms. Complete proteome coverage in the network was available for 37 species representing a wide phylogenetic range (S1 Table). No connections were initially established between nodes in this layer. Instead, we considered a third layer in which nodes (*V*_*F*_ *= {f*_*1*_,*f*_*2*_,*…}*) represented functional affiliation-type entities as nodes. These entities were Pfam domains [52], ortholog groups [53,54] and metabolic pathways [42]. We established links (E_PF_ edges) between layer-2 nodes (proteins) and layer-3 nodes (functional affiliation-type entities) based on current predictions derived from standard sequence analysis pipelines and annotation (see Methods). Lastly, we have used bioactivity data information to establish links (E_DP_ edges) between protein targets (layer-2) and chemical compounds (layer-1). These links were established after manual curation of the textual description of the assays, targets, and measured activities. Because bioactivities integrated into the TDR Targets resource contained also negative evidence (inactive compounds at relevant concentrations against a particular target), a significant amount of manual curation of these data was required for construction of the network. Therefore, E_DP_ edges in the final network graph represented sensible bioactivity information available for each protein target (bioactivity thresholds and criteria are described in Methods). A summary of the information and entities included in the network is available in Table 3.

**Table 3:** Composition of the Multilayer Network. G_0_ and G_1_ are the network graphs before and after applying the filtering criteria, respectively. The table lists the numbers of nodes in the three network layers (see Fig 1), and the edges connecting nodes within and across layers (see main text).

Multilayer Network Composition		
**Graph Nodes**	**G**_**0**_	**G**_**1**_
V_D_ (bioactive compounds)	1,488,034	1,487,919
V_P_ (proteins)	385,711	167,815
V_F_: All nodes	58,102	5,186
V_F_: Pfam domains	7,156	2,252
V_F_: Ortholog groups	50,779	2,789
V_F_: Metabolic Pathways	167	145
**Graph Edges**	**G**_**0**_	**G**_**1**_
E_DP_ (bioactivity links)	4,167,518	325.843
E_DD_: All edges	170,272,699	67,629,415
E_DD_: Similarity	44,403,424	44,402,716
E_DD_: Substructure	125,869,275	26,714,379
E_PF:_ All edges	738,682	718,277
E_PF:_ Pfam domains	333,188	331,928
E_PF:_ Ortholog groups	325,017	305,872
E_PF:_ Metabolic Pathways	80,477	77,389

Once the data was integrated in our network model, we proceeded to identify informative functional affiliation-type annotations that were relevant for drug discovery. Therefore, in the next step, we discarded 52,916 V_F_ nodes that were not linked to at least one *druggable* protein in our dataset (in this context “*druggable”* was defined operationally as a protein with at least one link to a compound in layer-1). The final resultant network comprises 2,252 informative affiliations to Pfam domains, 2,789 affiliations to ortholog groups, and 145 affiliations to metabolic pathways.

The second and third layers of the network defined, on their own, an affiliation or membership network, which is a special type of bipartite network [55,56]. An important feature of this kind of networks is that the inter-layer connectivity pattern can be used to infer intra-layer associations for each layer, via projection procedures [56]. In our case, adjacent links of shared functional affiliation nodes, *V*_*F*_, were used to define weighted links, *E*_*PP*_, between protein nodes, *V*_*P*_. These inferred edges condensed similarity information at the level of the biological and functional concepts contained in layer-3.

We have implemented two projection methodologies. In the first case we took into account a *relevance score*, *RS*_,_ for each affiliation node based on the statistical significance level of the over-representation of associated druggable proteins as obtained through a Fisher’s exact test (see Methods, an example is provided in Table 4). For the second alternative, in view of the broad degree distribution observed for affiliation nodes (see S1 Fig), we also considered an extra factor that relativized the score of large categories (see Methods for technical details). The rationale of this correction is to down-weight the contribution of very promiscuous annotation nodes (e.g. highly frequent protein domains such as the ATP-binding cassette, present in many functionally-unrelated protein families and orthologs). Although their presence helps to increase the connectivity of the protein network, it also skews the protein prioritization scoring and, as a general rule, favors specific kind of proteins towards the first places in the resulting rankings (see below).

**Table 4:** Weighting the functional affiliations of proteins based on their association to bioactive compounds. The table lists two examples of affiliation-type entities (Pfam domains), and their respective contingency tables used to evaluate the significance of association of proteins containing these Pfam domains to bioactive compounds, using Fischer’s exact test. Calculation of P-values was done for all affiliation-type entities in the network (Pfam domains, Ortholog groups, and metabolic pathways) using this methodology. Scores used to weight network edges were derived directly from P-values using a simple transformation (see Methods).

**Example 1. Affiliation entity: Pfam domain PF02931: Neurotransmitter-gated ion-channel ligand binding domain** (P-value: 4.33 10^−67^)		
	**Linked to active compounds**	**Not linked to active compounds**
**Proteins affiliated to this entity**	96 (25.9%)	275 (74.1%)
**All other proteins**	5,955 (1.80%)	325,453 (98.20%)
**Example 2. Affiliation entity: Pfam domain PF08441: Integrin alpha** (P-value: 0.09)		
	**Linked to active compounds**	**Not linked to active compounds**
**Proteins affiliated to this entity**	5 (8.5%)	54 (91.5%)
**All other proteins**	6,046 (1.82%)	325,674 (98.18%)

Taking into account either projection methodology, layer-2 and layer-3 could be collapsed into a single *protein-projected* directed and weighted layer (PP-layer, see Fig 1B). The PP-layer along with the original drug-layer (D-layer), defined a new graph1)1) *G*'(*V* = {*V*_*D*_, *V*_*P*_}, *E* = {*E*_*DD*_, *E*_*DP*_, *E*_*PP*_}) that allowed us to propagate drug-target information to address different drug-discovery problems as described below in the next sections. When necessary, we will note the resulting graphs as $G_{r}^{'}$ (projection using affiliation node’s relevance scores) or $G_{rk}^{'}$ (projection using relevance scores and penalizing high degree affiliation nodes) when the first and second projection methodologies were used, respectively.

### Target prioritization strategies

In this section we considered the problem of prioritizing targets from a query proteome ***Q*** for which compound bioactivity data is scarce or lacking altogether, as this is frequently the case for pathogens causing neglected tropical diseases. In this strategy we aimed to take advantage of the information contained in the network for other organisms to guide the prioritization of targets in our query species. The rationale of the approach relies on the assumption that relevant drug-target associations from other organisms, in concert with similarity relations between proteins (embedded in the *G’* network as *E*_*DP*_ and *E*_*PP*_ edges respectively) could be used to propagate meaningful associations through the network and therefore suggest novel drug connections for proteins in ***Q***.

To prioritize targets, we devised the following algorithm. First we identified the set of *druggable* targets in the *PP-layer* of network *G’*. These were protein nodes that were connected to at least one compound via an *E*_*DP*_ edge (e.g. protein cal.575054in Fig 1A). In the next step, these nodes were used as seeds for a neighbor voting scheme algorithm (*VS*) implemented over the PP-layer. As a result of this voting procedure, proteins in ***Q*** will receive a score which essentially is the weighted sum of all the *E*_*PP*_ direct links to seed nodes (i.e. known targets). See Methods for further details.

In order to illustrate the performance of this strategy we considered two query species ***Q*** each of which have known druggable targets: a mammalian proteome (***Q =*** *M*. *musculus*, often used as a model for human drug development), and a proteome from a protozoan parasite (***Q =*** *T*. *cruzi*, Chagas Disease). We deliberately chose a data-rich and a data-poor organism for this exercise to showcase the performance of the approach under these two contrasting situations. Whereas 8,429 *E*_*DP*_ edges involving 280 *V*_*P*_ nodes were present for *M*. *musculus*, only 319 *E*_*DP*_ edges were adjacent to 19 *T*. *cruzi* protein nodes.

The validation proceeds in each case by removing from the graph *G*, all *E*_*DP*_ bioactivity edges involving proteins of ***Q*** before projecting layer-3 into layer-2 and weighting *E*_*PP*_ edges. In this way, we ensured that no information extracted from the query organism was employed to build the two-layer *G’* network used to prioritize targets in ***Q***. After weighting and projecting the modified network graph, we assessed the performance of the prioritization strategy using Receiver Operating Characteristic (ROC) curves.

**Fig 2:**
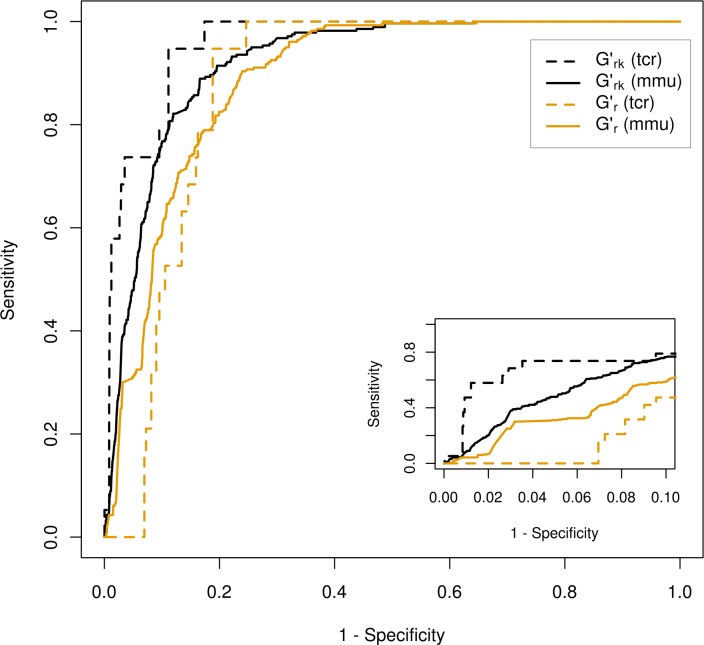
Cross validation procedure of the network-based target prioritization strategy. Receiver Operating Characteristic (ROC) curves for the recovery of Trypanosoma cruzi (TCR, solid black line) and Mus musculus (MMU, dashed yellow line) targets. 19 targets out of 6591 proteins and 280 targets out of 13575 proteins were considered for TCR and MMU respectively.

Fig 2 depicts ROC curves for predicted drug-target associations considering *G’*_*rk*_ (black) and *G’*_*r*_ (orange) for *M*. *musculus* (solid line) and *T*. *cruzi* (dashed line). Table 5 summarizes the performance of the prioritization procedures reporting the normalized AUC-0.1 values (see inset in Fig 2). The performance of a third prioritization strategy was also reported in the table for the sake of comparison. In this case, we considered a straightforward approach based on calculation of plain sequence similarity between druggable nodes in layer-2 against proteins in ***Q***. For this purpose we used the FASTA sequence-alignment tool [57], which produces longer alignments than BLAST (as it does not split the region of similarity into high-scoring-pairs as BLAST does).

**Table 5:** Performance of the network at the task of prioritizing targets (cross-validation). AUC-0.1 normalized scores (McClish Correction [58]) for different prioritization strategies based on voting algorithm over *G’*_*r*_ and *G’*_*rk*_ (Network Model) and a sequence similarity (alignment-based) methodology (FASTA). The statistical significance of the difference between *G’*_*rk*_ and FASTA AUC-01 values were evaluated through a 2,000 sample bootstrapping test and it is shown in brackets.

Organism	Network Model		FASTA
	G’r	G’rk	
*Mus musculus*	0.64	**0.72 (**2.8 10^−6^**)**	0.64
*Trypanosoma cruzi*	0.52	**0.81 (**8.1 10^−2^**)**	0.72

The high performance of our network model at the task of recovering the known targets in each organism reflects the fact that data from close relatives of both organisms are contributing substantially to the connectivity of these nodes in the network graph. As an example there are 60,540 E_DP_ edges connecting 455 V_P_ nodes in the case of rat data, whereas there are 43,325 E_DP_ edges connecting 3,567 V_P_ nodes for other protozoan and bacterial targets.

For both organisms, prioritizations based on the *G’*_*rk*_ network model presented the best performance. Down-weighting the relevance score of affiliation nodes by their degree provided a significant improvement, as prioritizations considering *G’*_*r*_ resulted in much poorer performances, especially for the *T*. *cruzi* case. Noticeably, the origin of the performance discrepancies between both network-based approaches were related to a strong correlation between prioritization scores in the *G’*_*r*_ network and the *strength* (a connectivity topological feature) of *V*_*p*_ nodes. This finding makes evident that *G’*_*r*_ prioritizations were *a priori* biased towards specific protein classes, i.e. those associated to high-strength *V*_*p*_ nodes (see Supplementary S1 Text).

It is worth mentioning that despite its simplicity, the voting scheme (*VS*) adopted for these network-based prioritization strategies has already proved to be competitive relative to more sophisticated algorithms in many scenarios, with the additional benefit of being extremely fast [59]. We verified that this was also the case in the context of our prioritization problem. In particular, we considered a prioritization strategy based on a network flow analogy (*functional flow* methodology) [60] and verified that it gave similar or inferior performance than *VS* (see S2 Table).

Finally, we compared the top ranked targets according to the network-based *VS* voting algorithm and the FASTA methodologies to see if the information provided by these alternative prioritization procedures were correlated. We considered the top 1% proteins ranked by the analyzed methodologies in each species (top 136 and 66 targets for *M*. *musculus*, and *T*. *cruzi*, respectively) (see S2 Fig). Even though we found statistically significant overlaps between *G’*_*rk*_ and FASTA predictions (Fisher Exact Test, p = 9.45 10^−28^ and p = 2.79 10^−2^ for *M*. *musculus*, and *T*. *cruzi*, respectively) most of these were specific to the considered prioritization strategy. This finding revealed that even if the two kinds of affiliation-type entities with the largest network coverage (i.e. orthology groups and Pfam domains) involved some sort of sequence similarity idea, the network based predictions were non-trivial from this point of view. Overall, these results also suggested that by considering different types of information in the network, we might gain alternative and complementary insights about potential targets for a query species.

### Prioritizing targets in kinetoplastid parasites

The most relevant and promising application of this kind of approach, is to prioritize new putative targets as interesting cases of study. To this end, we performed the procedure described above, hence taking advantage of the information contained in the network for known druggable targets across all species and analyzed the top ranked proteins for three kinetoplastids: *Trypanosoma cruzi* (TCR), *Trypanosoma brucei* (TBR) and *Leishmania major* (LMA) (the TriTryps [61]). The top 10 proteins resulting from this prioritization exercise are shown in the S3 Table. A detailed analysis of the candidate targets prioritized is not within the scope of this work. However, it is worth mentioning the finding of a number of interesting targets that have been already characterized in these parasites.

### Prioritization over $G_{r}^{'}$ (Non-normalized prioritization)

As shown in S3 Table, the majority of the proteins obtained at the top of the ranking using this kind of prioritization method were mostly protein kinases, one of the largest known protein superfamilies [62]. Apart from also being a rich source of highly druggable targets, from the point of view of the network this is a protein class with strong ties (abundant or heavy edges) between family members (both because of orthology and shared Pfam domains), and with abundant bioactivity links (E_DP_ edges) due to the recognized target promiscuity of kinase inhibitors [63].

The first protein in the ranking obtained for *Trypanosoma cruzi* was demonstrated to interact with and phosphorylate several parasite proteins [64], including some of the *trans*-sialidase family [65]. Transfection with a construct containing PKI (inhibitor of PKA) kills epimastigotes (genetic experiment), whereas treatment with the isoquinolinesulfonamide compound H89, a PKA inhibitor, killed 98% of the parasites within 48 hs (pharmacologic experiment) [64]. The 5th and 6th proteins obtained in the *L*. *major* and *T*. *cruzi* lists respectively is a casein kinase I isoform 2. This protein has been proven to be a target for 4 inhibitors in *L*. *major* [66]. These compounds also inhibited the growth of cultured *L*. *major* promastigotes and *T*. *brucei* trypomastigotes. In another work, the *L*. *major* protein was found to be inhibited by three 2,3-diarylimidazo[1,2-a]pyridines [67]. This target was also studied in *T*. *cruzi*, where it was found to bind the compound purvalanol B [68,69]. Finally, the *T*. *cruzi* protein obtained in 10th place of the ranked list, TcMAPK2, has been studied and characterized. Interestingly, this MAP kinase could not be inhibited by the mammalian ERK2 inhibitor FR180204, raising the possibility of a differential inhibition profile, which would open the door to the development of selective inhibitors of the trypanosome vs mammalian proteins [70].

### Prioritization over $G_{rk}^{'}$ (Degree-penalized normalization)

As shown in S3 Table, this kind of prioritization results in a more heterogeneous collection of protein classes at the top of the ranking.

The first protein in the prioritized list of *T*. *brucei* (listed 6th for *T*. *cruzi*) is an inositol 1,4,5-trisphosphate receptor. Inositol triphosphate receptors are intracellular calcium release channels that play a key role in Ca^2+^ signaling in cells [71]. Recent work in *T*. *brucei and T*. *cruzi* show that this target is essential for growth and establishment of infection [72,73]. The 3rd protein in the prioritized list of *T*. *cruzi* is a phosphatidyl inositol 3-kinase (PI3K). This protein has orthologs in several species and has 4 paralogs in humans. The PI3Ks can be divided into 3 classes (I-III). The protein prioritized by our method is a class I PI3K [74]. These enzymes are inhibited at nanomolar concentrations by wortmannin, which binds to the conserved ATP binding site of PI3Ks, suggesting that the drug could be active against all three PI3K classes. The PI3K pathway is also being investigated as target for intervention in cancer [74,75]. Given that our method identifies these proteins as potential target in parasites, this could present an opportunity to test promising molecules found in cancer research on the parasites. In *T*. *cruzi* the treatment with wortmannin, a PI3K inhibitor, prevented the entry of parasites to the cells [76,77]. A class III PI3K was recently characterized in this parasite and shown to be inhibited by wortmannin and LY294000 [78]. Another protein that appeared prioritized in our list (6^th^ for *L*. *major*, 9^th^ for *T*. *cruzi*) is the carbamoyl-phosphate synthetase II (CPSII), a key regulatory enzyme of the *de novo* pyrimidine synthesis. This enzyme, which generates carbamoyl-phosphate from L-glutamine, bicarbonate, and two ATP molecules, is the first in the 6-enzyme cascade that catalyzes the formation of uridine 5'-monophosphate. In a recent study, a CPSII knock out strain of *T*. *cruzi* displayed significantly reduced growth (in epimastigotes) [79]. Also, in fibroblast infection assays with metacyclic trypomastigotes, a smaller number of intracellular amastigotes were found in the case of infection with KO parasites. These results indicate that the *de novo* pyrimidine biosynthesis pathway and in particular this enzyme could be important targets to block parasite replication [79].

Another target suggested by this method is a lanosterol 14α demethylase (CYP51, 3^rd^in *L*. *major*, 5^th^in *T*. *brucei*). This finding represents a special case that serves both to validate the strategy and to highlight a number of gaps in the data curation process (see also Discussion). CYP51 enzymes belong to an ortholog group that contains 72 sequences, including human and trypanosomatid sequences. This protein is a cytochrome P450 that in fungi and kinetoplastid protozoa catalyzes a key biochemical step in the ergosterol biosynthesis pathway [80]. The enzyme is a known validated target for chemotherapy against *T*. *cruzi*. However, a careful analysis of the prioritized lists revealed a clear gap in the availability of curated bioactivity data: the *T*. *cruzi* enzyme was the only trypanosomatid ortholog in the network that was linked to bioactivity data (and therefore our algorithm considered it as a *seed* target, and accordingly, the *T*. *cruzi* enzyme was not present in the final prioritized list). But a number of studies have already reported on the inhibition of the *T*. *brucei* and *Leishmania* enzymes with CYP51 inhibitors [81–83]. However, these data were not present in the TDR Targets and/or ChEMBL releases used to build the network. Therefore, these targets have been prioritized under the assumption that no bioactivity information was available. In this case, the target suggestions made by the network only served to identify these gaps, because the experimental work required to validate these targets and their inhibitors was already present in the literature.

### Proposing candidate targets for orphan compounds: Strategy

In drug discovery it is often the case that high-throughput phenotypic screenings are conducted on whole organisms or whole cells in culture. This is a good strategy to filter large libraries and identify reasonable "hit" compounds. However, to develop these compounds further it would be advantageous to know the target(s) of the compound, to gain an understanding of the mechanism of action of the drug.

In this part of the work we took advantage of the information contained in the constructed network to obtain candidate targets for a given orphan compound, defined as a node in the D-layer of our network with no links to the PP-layer. We assume that these compounds have been selected based on one of the case scenarios described above (i.e. from high-throughput phenotypic screenings). Such compounds (here referred to as “orphan molecules” ***m***) have no links to the PP-layer but have bioactivities that meet the different specified cutoffs in Table 2 In these cases, we are interested in getting a prioritized list of putative targets for each orphan molecule ***m***. For this, we only report here results obtained considering the *G’*_*rk*_ network-based strategy, as the already observed bias for the *G’*_*r*_ network-model affects the sensitivity of the corresponding prioritization results as shown in previous sections.

We first proceeded by identifying the chemical similarity neighborhood of ***m*,** *CSN(m)*, taking into account molecules directly linked to ***m*** through *E*_*dd*_ edges. Next, we considered the set of target proteins in the PP-layer that were associated to the *CSN*(***m***) through bioactivity annotations. These protein nodes were used as seeds for the prioritization procedure described in the previous sections. Each seed protein, *s*_*j*_, was associated to an initial score, *w*_*j*_ (see Eq(7)) proportional to the overall chemical similarity reported between *CSN*(***m***) and the considered orphan compound of interest ***m*** (see Methods).

To validate this strategy, bioactive molecules with known targets were artificially “orphaned” by removing the bioactivity links that associated these drugs with their cognate targets. We considered a random set of 1,000 molecules (out of ~10^5^) with exactly one known protein target in our dataset, and assessed our ability to recover these targets in the prioritized lists after removing the corresponding bioactivity links.

Under this cross-validation exercise, we first proceeded to analyze the global sensitivity of our recovery strategy. For each artificially orphaned drug ***m***, we computed both a global ranking, *r*_*G*_, of putative target proteins from all available organisms in the network, and a species-specific ranking list, *r*_*SS*_, where the prioritized proteins come only from a single organism (in this case the source of the original target).

**Fig 3:**
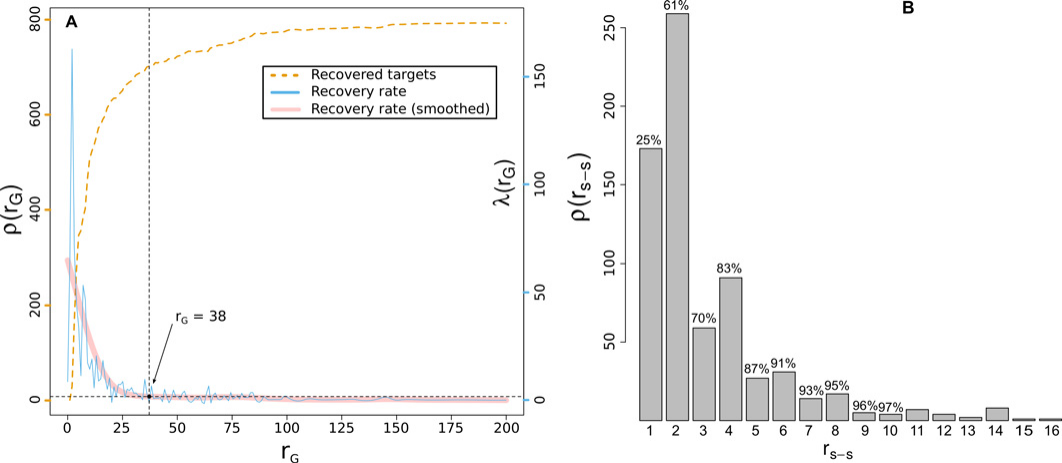
Performance at the task of recovery of the correct target for artificially orphaned compounds. **A:** Number of recovered proteins, *ρ*(*r*_*G*_) (left scale, blue line), and protein recovery rate *λ*(*r*_*G*_) (right scale, orange dashed line) as a function of global ranking threshold values, r_G_. The horizontal black dashed line represents 3 standard deviations (3σ) above the mean asymptotic noise level (see text). **B**: Distribution of species-specific ranking positions, r_ss_, for the 703 recovered true-targets which presented global ranking values lower than r*_G_ = 38 estimated in panel A. Cumulative fraction of recovered targets is shown above bars.

The plot in Fig 3A shows, for different thresholds *l* of the global rankings *r*_*G*_, the number of recovered targets, ρ(*r*_*G*_), and the corresponding recovery rate, λ(*r*_*G*_), defined as the ratio between the incremental gain in *ρ*, per ranking interval (i.e. $\lambda \left(r_{G}=l\right)=\Delta \rho \left(r_{G}\right)\;/\;\Delta r_{G}{|}_{r_{G}=l}$. In addition we found it useful to consider a third-order spline approximation, $\tilde{\lambda }(r_{G})$ to smooth out rapid fluctuations of *λ*(*r*_*G*_).

As can be appreciated in Fig 3, the recovery rate of the original target for each compound, $\tilde{\lambda }(r_{G})$ $\tilde{\lambda }(r_{G})$, rapidly drops converging to an asymptotic value near zero. This suggests that increasing the number of prioritized targets (e.g. the prioritization list length) above a given global ranking position gives on average no significant increment in the number of original targets recovered. We estimated the asymptotic recovery rate level, *λ*_∞_, as the mean $\tilde{\lambda }$ value obtained disregarding the first 50 ranking positions, and estimated the corresponding noise level, σ, as the variance of the corresponding $\tilde{\lambda }$ values. Taking into account these quantities, we further defined an optimal list length $l=r_{G}^{*}$ for which the recovery rate was significantly higher than the asymptotic value:

$${\text{r}}_{G}^{*}=\underset{l\epsilon [1,1000]}{\text{max}}\left\{\tilde{\lambda }\left(l\right)\geq \lambda_{\infty }+3\sigma \right\}$$ (9)

This parameter serves to identify a global ranking range (i.e. the r*_G_-top ranked molecules) where reasonable predictions can be anticipated, in the sense that a high rate of success is expected to occur. In our cross-validation study we found that r*_G_ = 38. Considering this threshold level, the sought target proteins were globally ranked before r*_G_ for ~70% of the 1,000 tested molecules. Fig 3B shows how these 703 targets were ranked according to the corresponding species-specific ranking lists (*r*_*SS*_). We observed that 70% of these predicted target proteins appeared at the top three positions of the corresponding *r*_*SS*_ ranking, and ~97% were ranked within the top 10 suggested targets. On the other hand, we observed that top-ranked target proteins for 297 out of the 1,000 tested molecules were globally ranked after the $r_{G}^{*}$ position. For these cases we assumed that the information embedded in the network was not enough to successfully recover the original targets, as even the best predictions for the corresponding organism laid on a twilight-zone of the algorithm suggestions given the adopted threshold level. The considered threshold of 3σ, although arbitrary, represented a sensible value because, as shown in Fig 3B, the corresponding global ranking threshold, r*is found within a sharp change of regime (i.e. an elbow) of the recovery rate curve.

In summary, our methodology was able to retrieve the correct association within experimentally affordable prioritization lists for 70% of the artificially ‘orphaned’ compounds. Noteworthy, we also introduced a metric based on the performance of recovery tasks of artificially orphaned compounds, to recognize problematic species-specific prioritization scenarios.

**Fig 4:**
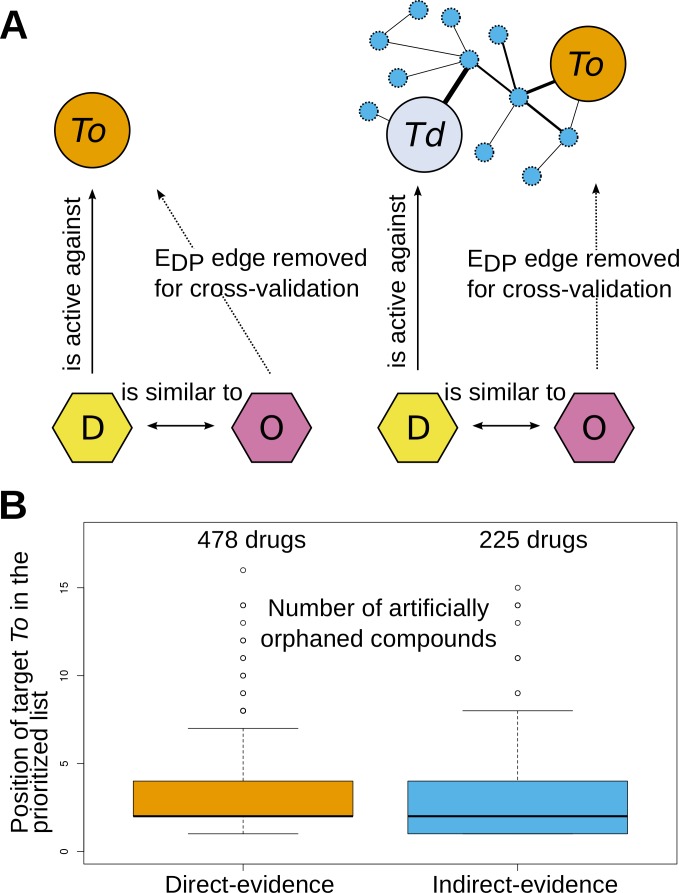
Inference of targets of orphaned compounds. **a, top:** Schematic view of two different ways in which the algorithm can find the correct target for artificially orphaned compounds. O = orphan compound; D = bioactive drug/compound which is a first neighbor of O in the D-layer; T_o_, known target of the artificially orphaned compound O; T_d_, known target of compound D. Arrows represent significant similarity relationships between compounds or significant bioactivity links between a target and a compound. Dashed lines connecting compounds and targets represent the original E_DP_ edges that were removed for the cross-validation procedure. **a, left:** direct inference, compound D has a bioactivity link to T_o_ (special case, T_o_ = T_d_). **a, right:** indirect inference, compound D lacks bioactivity links against T_o_, but a high-scoring path connects T_d_ to T_o_ in the projected PP-layer. **b, bottom:** boxplots showing the distribution of the position of T_o_ targets in the rankings for 703 orphaned compounds. **b, left:** boxplot for cases that fell in the direct inference class (478 compounds). **b, right:** boxplot for cases in the indirect inference class (225 compounds).

Finally, we found it informative to analyze the way in which we were able to recover the original target in this exercise. As shown in Fig 4 there are essentially two ways in which we can guess the target of an orphan compound. The first is through a very short path in the network (leftmost panel in Fig 4A), that directly connects the orphan compound with a bioactive compound that is in turn linked to the original (artificially orphaned) target. This was the case for 478 (68%) of the 703 recovered targets. However, in 225 cases (32%) the recovered target lacked direct bioactivity links to molecules that were neighbors of the orphan compound in the D-layer graph. In these cases, the corresponding target could not have been recommended without the adopted network approach (rightmost panel in Fig 4). These results thus show that the network contains redundant information that can still suggest the correct targets, with high specificity in the absence of direct bioactivity links. This performance suggests that our network model can be useful as an aid to propose experimental studies on orphan compounds.

### Proposing candidate targets for orphan compounds: Application to *Plasmodium falciparum*

As a case study, we used the network to infer targets for compounds which presented significant activity against *Plasmodium falciparum*, but that did not appear listed in target-based assays in our dataset. There were 19,124 compounds derived from a number of recent high-throughput screenings against *P*. *falciparum* [29–31]. From this dataset, 9300 molecules were amenable to our prioritization methodology, as they had at least one neighbor drug presenting bioactivity on at least one protein target. Using the strategy described in the previous section, we were able to suggest candidate targets for 176 of these compounds when r*_G_ *= 38* (see S4 Table).

**Fig 5:**
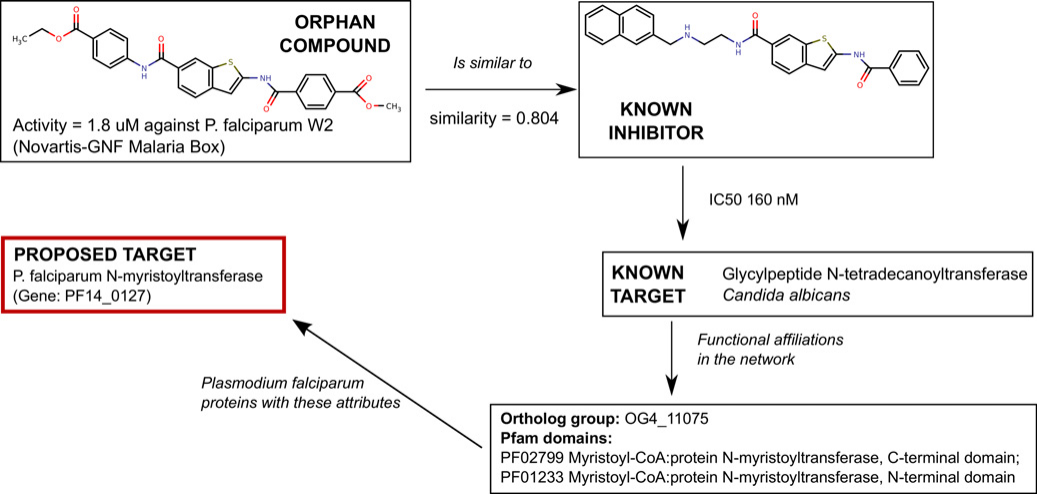
Suggesting targets for orphan compounds: example 1, N-myristoyltransferase. The compound shown in the upper panel (TDR Targets ID 606689, ChEMBL ID 688510) is an orphan compound (no known target) that was shown to be active against P. falciparum. A similar compound (Tanimoto similarity coefficient = 0.804), shown at the right, is active against a glycylpeptide N-tetradecanoyltransferase of Candida albicans [78] which belongs to the same ortholog group, and shares 2 Pfam domains with the P. falciparum N-myristoyltransferase (PlasmoDB ID PF14_0127).

One example of this drug-target prediction is shown in Fig 5. The orphan compound shown in the figure (a benzothiazoline) was found to be active against *P*. *falciparum* strain W2. However its mechanism of action is currently unknown. In our network, the connectivity map of this compound, leads to the N-tetradecanoyltransferase of *C*. *albicans*. This enzyme catalyzes the N-myristoylation of proteins, in which a myristate molecule (14-C saturated fatty acid) is added to the N-terminus of a glycine residue in specific target proteins [84,85]. We validated our prediction by doing *a posteriori* analysis of the literature. First, several studies show that this protein is indeed a promising target for development of new antimalarials [86–88]. Furthermore, a number of benzothiazole compounds have already been tested against the *Plasmodium* enzyme [88]. Interestingly, none of the compounds reported in these papers were part of our dataset, and therefore were not included in our network model (see Discussion on data curation gaps below). Therefore, though similar, both the orphan compound, and the compound that has been shown to inhibit the *C*. *albicans* enzyme are different compounds.

**Fig 6:**
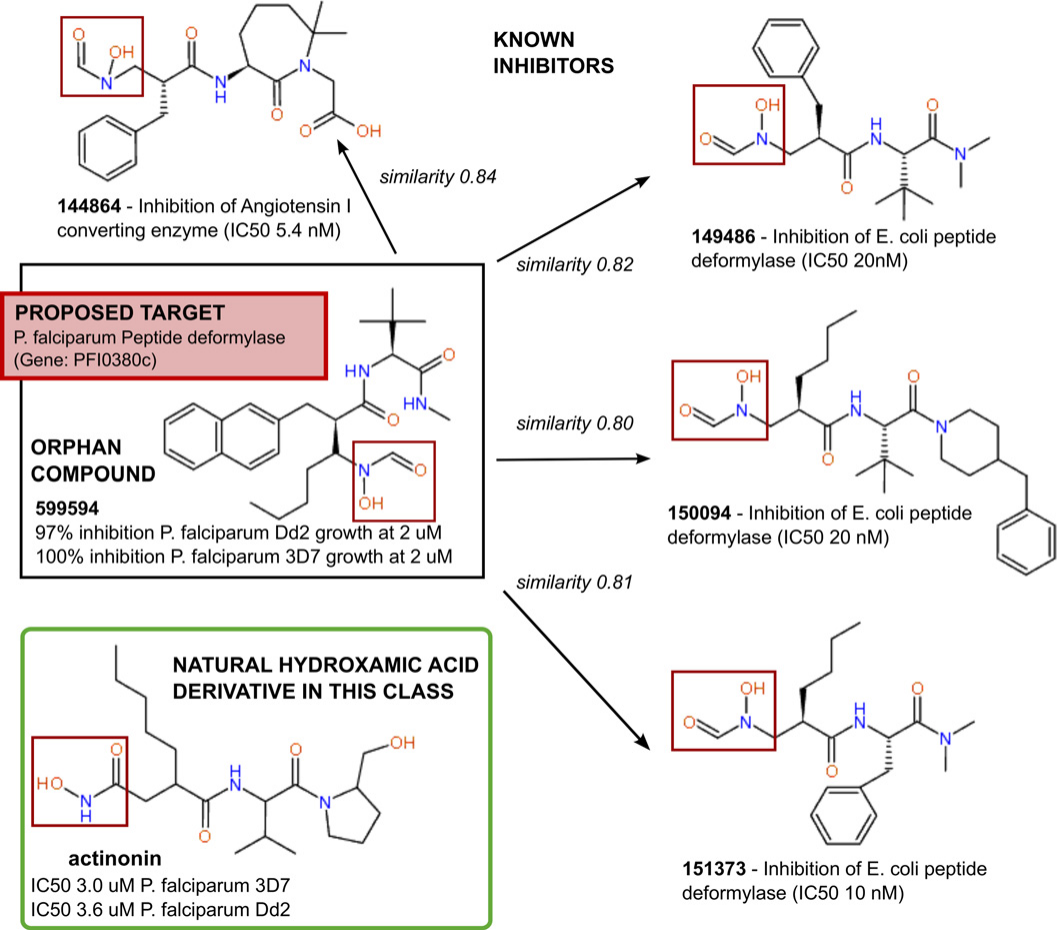
Suggesting targets for orphan compounds: example 2, peptide deformylase. Following the strategy described in the text, and visualized in Figs 4 and 5, based on the functional affiliations of the chemical similarity neighborhood of orphan compound 599594 (TDR Targets ID), the target of this compound is proposed to be a peptide deformylase.

Another interesting case is shown in Fig 6. In this case the orphan compound (TDR Targets ID 599594) [29] was shown to be active at 2 μM against the wild-type *P*. *falciparum* strain 3D7 and the multidrug-resistant strain Dd2 (100% and 97% growth inhibition, respectively). In our network model this compound is connected with other active compounds, with varying levels of similarity, as shown in the figure. All these compounds are hydroxamic acid derivatives, some of which are known to inhibit bacterial peptide deformylases [89]. The most frequently used inhibitor of peptide deformylases, actinonin, was also shown to be active against *P*. *falciparum* [90], as well as other hydroxamates [91]. Although it remains to be seen if these orphan compounds are active against this enzyme, or if they hit other cellular targets (compounds containing the hydroxamic acid moiety often possess a wide spectrum of biological activities [92]), this example serves to highlight the types of target/chemical hypotheses that our network model generates. As mentioned above, the best candidate target from *P*. *falciparum* for this orphan compound was ranked in the prediction zone, under 3σ (r*_G_ < 38).

**Fig 7:**
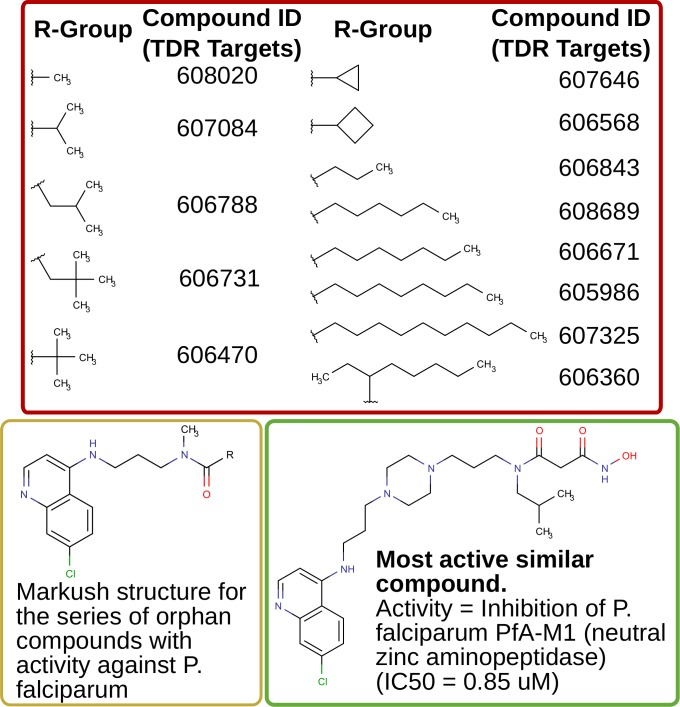
Suggesting targets for orphan compounds: alanyl aminopeptidase. A series of 13 structurally related orphan compounds (EC50 values range = 0.03–0.74 uM against *P*. *falciparum*, *data from* [50], available at TDR Targets) are connected in our network model to the PfA-M1 *Plasmodium* enzyme. In the figure we show the Markush structure for the series, and the corresponding R-groups and their database IDs. We also show one representative of other active similar compounds (see [95]) with activity against defined targets in the network.

Other orphan compounds with antimalarial activity (Fig 7) were connected in our network model to a *Plasmodium falciparum* M1 alanyl aminopeptidase (PfA-M1). This enzyme has been shown to be an essential hemoglobinase, catalyzing the final stages of hemoglobin break-down within intra-erythrocytic parasites [93,94]. A number of inhibitors have been described for PfA-M1 [95–98], and some of these have been shown to control both laboratory and murine models of malaria [97]. In our network model, some of these inhibitors are part of the chemical similarity neighborhood of a series of structurally related orphans (shown in the figure).

**Fig 8:**
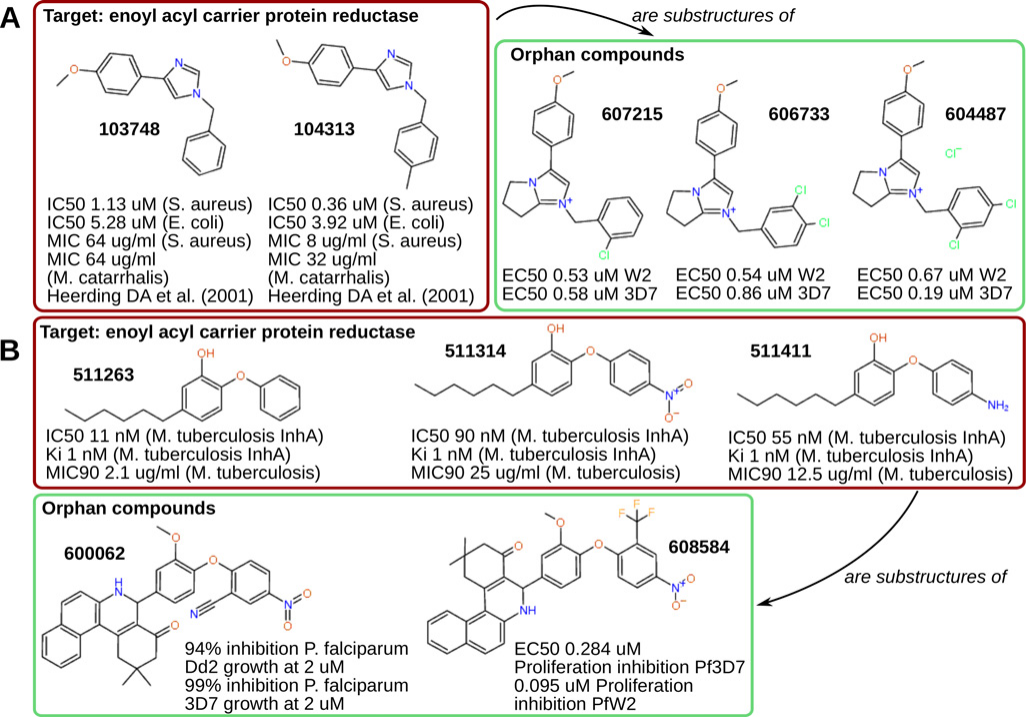
Suggesting targets for orphan compounds: enoyl-acyl carrier protein reductase. Two groups of orphan compounds (panels A, B) with structurally different scaffolds are linked to inhibition of enoyl-acyl carrier protein reductases.

Five orphan compounds (Fig 8) where proposed to act through the enoyl-acyl carrier reductase (FabI). This enzyme is involved in fatty acids biosynthesis type II, a pathway that is essential for correct liver stage parasites development [99]. FabI has been validated as drug target for antibacterials and antimalarials, such as triclosan, a drug that inhibits this enzyme in several species, including *E*. *coli*, *M*. *tuberculosis*, *S*. *aureus* and *P*. *falciparum* [100,101]. Several other compounds have been tested recently as potential inhibitors of this target in *P*. *falciparum* [99,102–104] and in other parasites [105]; however the suggestions made by our network model constitute novel hypotheses.

In some other cases, the compounds had proposed targets that, to our knowledge, have not yet been characterized experimentally as potential drug targets in *P*. *falciparum*. This is the case of a putative 3-demethylubiquinone-9 3-methyltransferase (PF3D7_0724300), a putative 3-oxo-5-alpha-steroid 4-dehydrogenase (PF3D7_1135900), and a putative polyprenol reductase (DFG-like protein, PF3D7_1455900) [106]. An exception is perhaps the putative glycerol-3-phosphate acyltransferase (LPAAT, PF3D7_1444300), an ortholog of which was recently validated as an essential gene for blood stage replication in a murine Malaria model [107]. The bioactive orphan compounds shown in S4 Table therefore can serve as potential starting points to explore the chemical space around these targets.

## Discussion

In this work we show a novel multilayer network strategy that addresses a number of important problems in the field of drug discovery as applied to neglected tropical diseases. First, we show how the information integrated in a multilayer network containing complete proteomes from pathogen and non-pathogen organisms allow the identification of relevant candidate drug targets, even in the presence of scarce target inhibition data for the pathogen of interest. This is particularly important in this field as this provides a mean to leverage data from other, more studied organisms to guide drug repositioning exercises for diseases that usually lack experimental, high-volume, chemogenomic datasets.

### On different prioritization strategies

We and others have previously devised a number of target-centric prioritization strategies that were focused on target features with only minor integration of chemical information [26,27,108,109]. In these prioritizations, targets were assigned scores based on *a priori* defined sets of criteria by different users and different *ad-hoc* scoring systems for target features. In contrast, in this work we show how the availability of target-drug associations in our network model (E_DP_ edges, derived from curated bioactivity assays) can be used to guide the scoring of targets (weighting of graph edges) through a simple statistical assessment of enrichment of seed proteins (known targets of bioactive compounds) for functional annotation classes (target features), followed by prioritization of first-neighbors using a voting algorithm. As a result, we are now able to prioritize targets without resorting to *ad-hoc* hypotheses about desirable or undesirable target features.

The network model (when normalized affiliation relevance scores were considered) showed an increased performance when compared to a simple (naïve) sequence similarity search against known druggable targets, (Table 5).

Moreover, our methodology provides additional flexibility as two different graphs, *G’*_*r*_ and *G’*_*rk*_, can be derived from the original network to perform prioritization tasks. Differences in the respective ranking lists could be understood in terms of the observed prioritization dependency on the *strength* of target nodes in the *G’*_*r*_ graph. The *strength* of a node in a weighted graph takes into account not only its degree (i.e. the number of connections to other adjacent nodes) but also the weighted values of these connections. As discussed in detail in Supp. Text S1, prioritizations based on uncorrected scores (*G’*_*r*_ network) were *a priori* heavily driven by strong nodes. A bias towards these high-strength nodes may not be necessarily bad, as the strength reflects embedded information on functional categories enriched in links to active compounds (initial score or weight of a seed node. In the particular case of prioritizations derived from the G’r graph, the high enrichment in targets from the highly druggable protein kinase superfamily may be a desirable outcome. In spite of this, host toxicity and inhibitor promiscuity are potential concerns in this case, as this is the largest family of druggable targets that binds to a common substrate (ATP) with numerous examples of inhibitors targeting several kinases at low micromolar concentrations [63].

Additionally, when considering the prospects of testing the compounds associated with these targets in whole-cell assays against other organisms, it is worth considering that perhaps because of this demonstrated promiscuity, there have been many cases of success in the identification of non-kinase targets of kinase inhibitors [110–114]. This provides a counter example of the utility of these highly biased G’r prioritizations.

Finally, as shown in S2 Fig, there is a negligible overlap between the sets of recovered targets following each strategy. This result highlights the complementarity nature of the different explored prioritization methodologies suggesting that by considering different types of information, we might gain alternative and complementary insights about potential targets for a query species.

### On finding targets for orphan compounds

Prediction of candidate targets for orphan compounds is not straightforward. Several approaches rely on chemical similarity to relate ligands to candidate targets [17,18]. However, this type of similarity-based strategies can only provide starting points that should be further validated experimentally. It is well known that only a fraction of chemically similar compounds (Tanimoto coefficient > 0.85) are active against the same given target [49]. Furthermore, some compounds are able to modulate several targets [115,116], introducing another layer of complexity. In our case we have taken advantage of the integrated data to connect protein targets to bioactive compounds that lack target-based assay information. Inspired by how medicinal chemists search for putative targets, we have done this by essentially prioritizing targets that are connected to the “chemical similarity neighborhood” of orphan compounds. However we believe our approach improves over current methods for deorphanizing compounds by i) doing this in an automated and unified way (e.g. applying the same rules and parameters for all compounds) at a large scale; and ii) introducing a different approach when identifying candidate pathogen targets by using a combined metric that results from the projection of 3 functional features instead of solely relying on sequence similarity (e.g. as in the FASTA approach we performed for comparative purposes). Moreover we have introduced a data-driven methodology to identify *a priori* reliable species-specific rankings, given observed global ranks of protein targets along the entire network. Some of the connections highlighted by our model were supported by independent experimental validations as found *post-facto* in the literature. However, further experimentation should be carried out to test the activities of other orphan compounds (and their analogs). In this context it is appropriate to bear in mind the high attrition rate that is usually associated with confirmatory assays, even when performing these on the very same pathogen species [117].

The utility of this approach lies not only in the search for new chemical leads for drug discovery, but also to identify and map tool/probe compounds [118,119]. Although good drugs and good tool compounds must meet different criteria [118,119], we argue that particularly for neglected tropical diseases, integrative approaches that help leverage any available chemical information for advancing basic research would also have an impact in the long term in the drug discovery process. In this sense, by providing connections between orphan bioactive compounds and putative targets, our network model has the ability to propose new testable hypotheses.

### Problems and caveats identified revolve around data curation

As part of this work we have identified some significant gaps in the curation of bioactive compounds. When looking for recent reports that could serve as a *post-facto* validation of our findings, we noticed a number of publications with relevant information but that pre-dated the initial data gathering exercise for this paper (see Results). These represent a set of papers that passed unnoticed to a number of curation efforts. One example is the paper by Bowyer *et al* published in 2007 in which the authors show that a number of benzothiazoles were active against *P*. *falciparum* NMT [88]. Because these compounds were not present in our data sources, they were not included in our network model. Luckily for us, they could be used to independently validate the proposed target for one of our orphan compounds (see Fig 5). However, and perhaps more importantly, this case also helps to raise awareness of the ever important problem of manual curation of data present in the literature.

Construction of our network model also required some manual curation, which represents a huge bottleneck in terms of time invested at this task. The single most laborious step in our approach has been the manual curation required to classify compounds into active vs inactive. This was necessary because bioactivity databases such as ChEMBL include negative data as well (e.g. curated data for all *assayed* compounds). However, upon detailed scrutiny, the disparate ways and units in which bioactivities are reported (IC50s, EC50s, Kis, %inhibition, etc.) demanded a serious and very time consuming curation effort. This is the main reason limiting the number of links between the D-layer and the PP-layer in our network model. Adding more proteomes (and calculating their annotation-type affiliations), or more compounds (and calculating their substructure and similarity relationships), is just a matter of throwing more computational resources at the problem. However, increasing the number of links between targets and compounds still requires a heavy investment in data curation.

Another critical issue in our network model that was directly related to this data curation gap was the definition of active vs inactive compounds in cases where the activity of a compound was reported as a relative measure (e.g. a percentage) of a defined outcome. We have decided to use 80% activity as a cutoff (see Methods), but we are aware of many examples in the TDR Targets and ChEMBL databases where activity >80% is due to compounds tested at concentrations that exceed reasonable or physiological concentrations. But because this information is present in the textual descriptions of the assays (and not as part of a separately queryable field), either a big investment in manual curation or in the use of natural language processing of these data is required to further extract and correct for these cases. During data curation we accepted all compounds with >80% activity, in whatever assay was performed, and we only checked the concentrations of the inhibitors used in a case by case basis for the examples shown in the figures.

### Future prospects

The network model developed in this work can certainly be expanded further, connecting more targets from other proteomes of interest, and connecting more compounds. We have already identified recent datasets listing bioactivities of new and existing compounds (DNDi Chagas and Human African Trypanosomiasis screenings, GSK TCAMS Tuberculosis and Chagas HTS, among others). These are already in the public domain [45,120]. We are also working to expand the TDR Targets resource to include more pathogen genomes, including a number of helminths causing important human diseases, such as *Echinococcus spp*. (Hydatid disease) [121], *Loa loa* (loiasis) [122], Fasciola hepatica [123], and other protozoan pathogens such as *Trichomonas vaginalis* [124] and *Giardia* [125,126]. This would allow scientists interested in these pathogens to take advantage of the integrated chemogenomics datasets in the network to prioritize candidate targets and compounds for these diseases.

Finally, although theoretically the model can also be expanded to include other types of affiliation-type annotations, or relations, these would have to be amenable to obtain from scalable computational analyses, in order to avoid the curation bottleneck. For example, one of the most valuable query types supported by TDR Targets is based on integration of phenotypic annotations (e.g. ‘*is the target essential for the cell*?’). These functional genomics data are mostly derived from genome-wide experiments (knockouts or knockdowns). However, it takes a sustained curation effort to identify, and integrate these data for all the genomes of interest.

### Conclusion

Our network model provides a way to query large chemogenomics datasets by integrating data from both phenotypic and target-based screening strategies. As a result, we enable a cohesive view of these different approaches to drug discovery. Once built, the network can sustain fast queries on these diverse data types and a simple rationalized navigation through the connected drug-target space.

## Supporting Information

S1 Fig Degree distribution for affiliation nodes. The plot shows the distribution of the number of associated proteins to a given affiliation node.
(TIFF)

S2 Fig Overlap between top-ranked targets according to different prioritization criteria. Overlap between top ranked proteins (1%) according to G’r, G’rk and FASTA (naïve, sequence similarity only) strategies is shown using Venn diagrams for two genomes: *M*. *musculus*, and *T*. *cruzi*.
(TIFF)

S3 Fig Filtering substructure relationships for promiscuous molecules. The plot shows the number of compounds involved in substructure similarity relationships that can be filtered out as a function of molecule size (MW) for different promiscuity threshold levels (different curves). PS = parental structures (those that contain a compound as part of its structure).
(PDF)

S4 Fig Performance dependence of cross-validation experiments on the free parameter α. The figure shows the performance of 10-fold cross-validation target prioritization exercises in which all target-compound bioactivity links were removed for two query species (*T*. *cruzi* and *M*. *musculus*). Network projections were calculated using different values of the free parameter α. Overall we observed that differences in AUC-0.1 values were within 5% of tolerance.
(TIFF)

S1 Table List of organisms with complete genomes included in our network model. We list the name of the organism and a brief summary of the taxonomic classification or grouping for each species.
(PDF)

S2 Table Comparison between proposed prioritization network methods. Voting Scheme (VS) and Functional Flow (FF) network prioritization strategies were compared in terms of its AUC-0.1 performance (normalized scores were reported by using McClish Correction [56]). In spite of the more sophisticated procedure, FF performance did not improve the VS simpler performance. In the table we also show AUC-0.1 values corresponding to alternative versions of our affiliation network in which we removed one type of functional affiliation in each case. S = score used to weight *E*_*PF*_ edges in the network (see main text); K = degree of each *V*_*P*_ node; S/K = normalized score over the degree. In both cases, S/K network outperform performances.
(PDF)

S3 Table Top kinetoplastid proteins ranked in a network-based prioritization. Targets were prioritized using either non-normalized scores (Sheet "NN Prioritization"), or after applying a degree-normalizing scoring function (Sheet "DN Prioritization"). Three rankings are shown in a single table in each spreadsheet (T. cruzi, L. major, T. brucei). The first column therefore shows the corresponding position of the target in each ranking. For simplicity, the affiliation of targets to metabolic pathways is summarized in an EC number. Ortholog groups (OG) are OrthoMCL IDs, Target IDs are either from TriTrypDB (prioritized targets) or ChEMBL (druggable homologs). For clarity, a single representative druggable homolog is shown. In the non-normalized prioritization, because the list is composed mostly exclusively by protein kinases, two additional columns are used to provide information on their classification, according to Bahia et al. [71].
(XLSX)

S4 Table Complete list of putative targets for orphan compounds that are bioactive against *P*. *falciparum*. (XLSX)

S1 Text Relevance scoring scheme and predictive power. We analyzed in detail the discrepancies among *G’*_*rk*_ and *G’*_*r*_ based prioritizations. We showed how the performance discrepancy happened to be related to a strong correlation that existed between *G’*_*r*_ -prioritization scores and network´s connectivity topological features.
(PDF)
