# Supplementary figures and images for: A Multilayer Network Approach for Guiding Drug Repositioning in Neglected Diseases
Source: PLoS Negl Trop Dis. 2016 Jan 6;10(1):e0004300. doi: 10.1371/journal.pntd.0004300 (PMC4703370; doi:10.1371/journal.pntd.0004300)

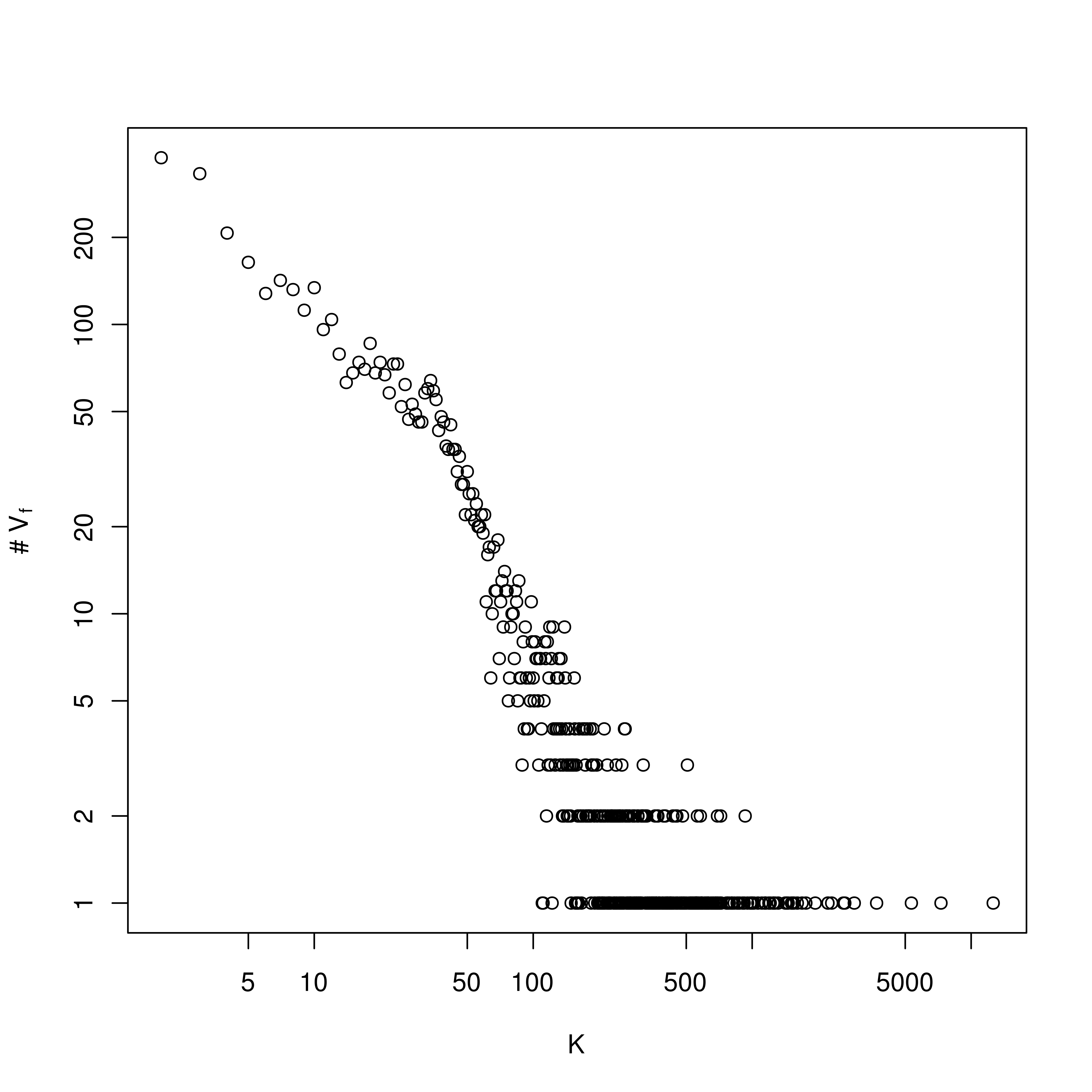

Supplement: S1 Fig — The plot shows the distribution of the number of associated proteins to a given affiliation node. (TIFF) [file pntd.0004300.s001.tiff]

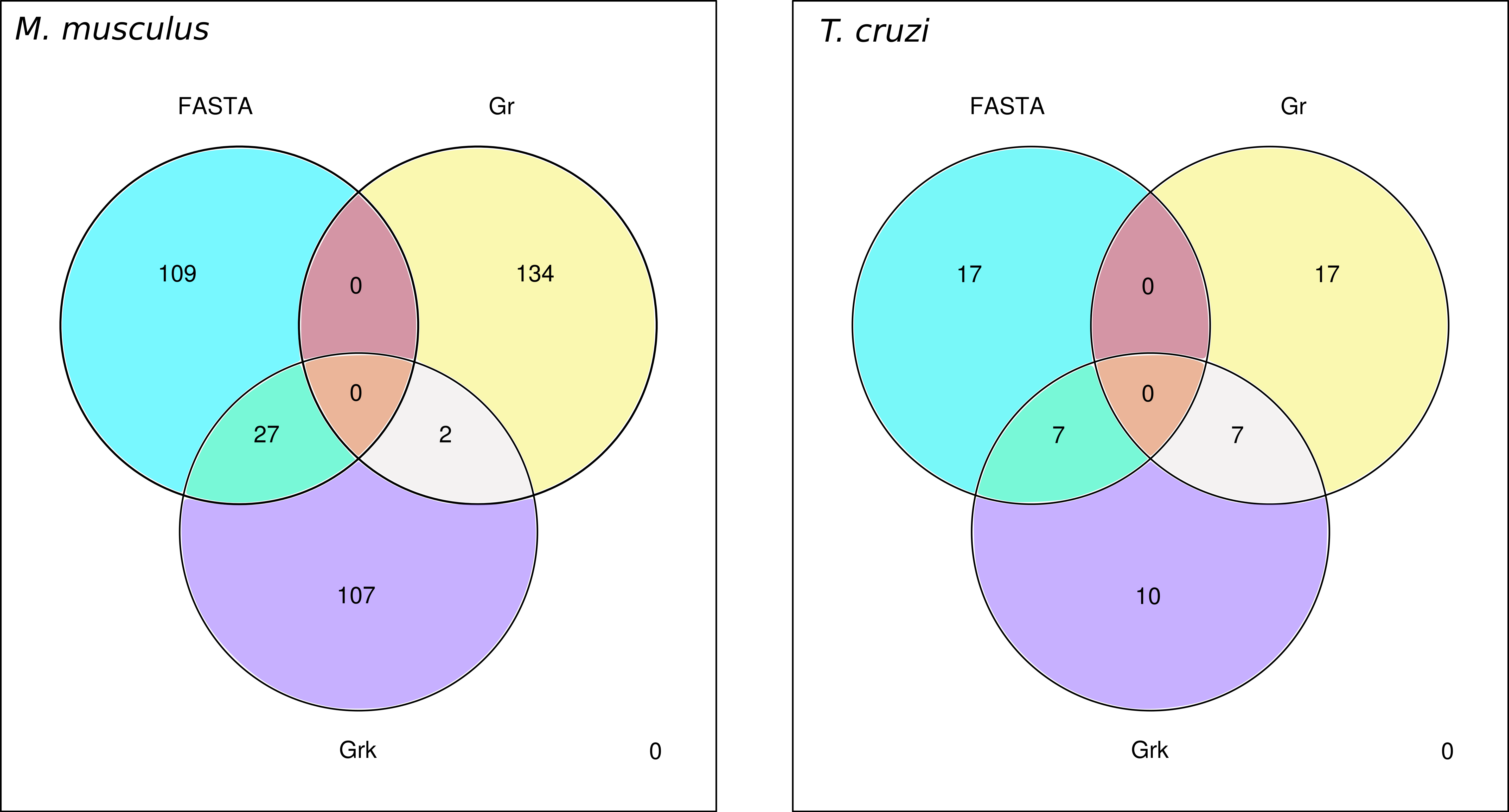

Supplement: S2 Fig — Overlap between top ranked proteins (1%) according to G’r, G’rk and FASTA (naïve, sequence similarity only) strategies is shown using Venn diagrams for two genomes: M. musculus, and T. cruzi. (TIFF) [file pntd.0004300.s002.tiff]

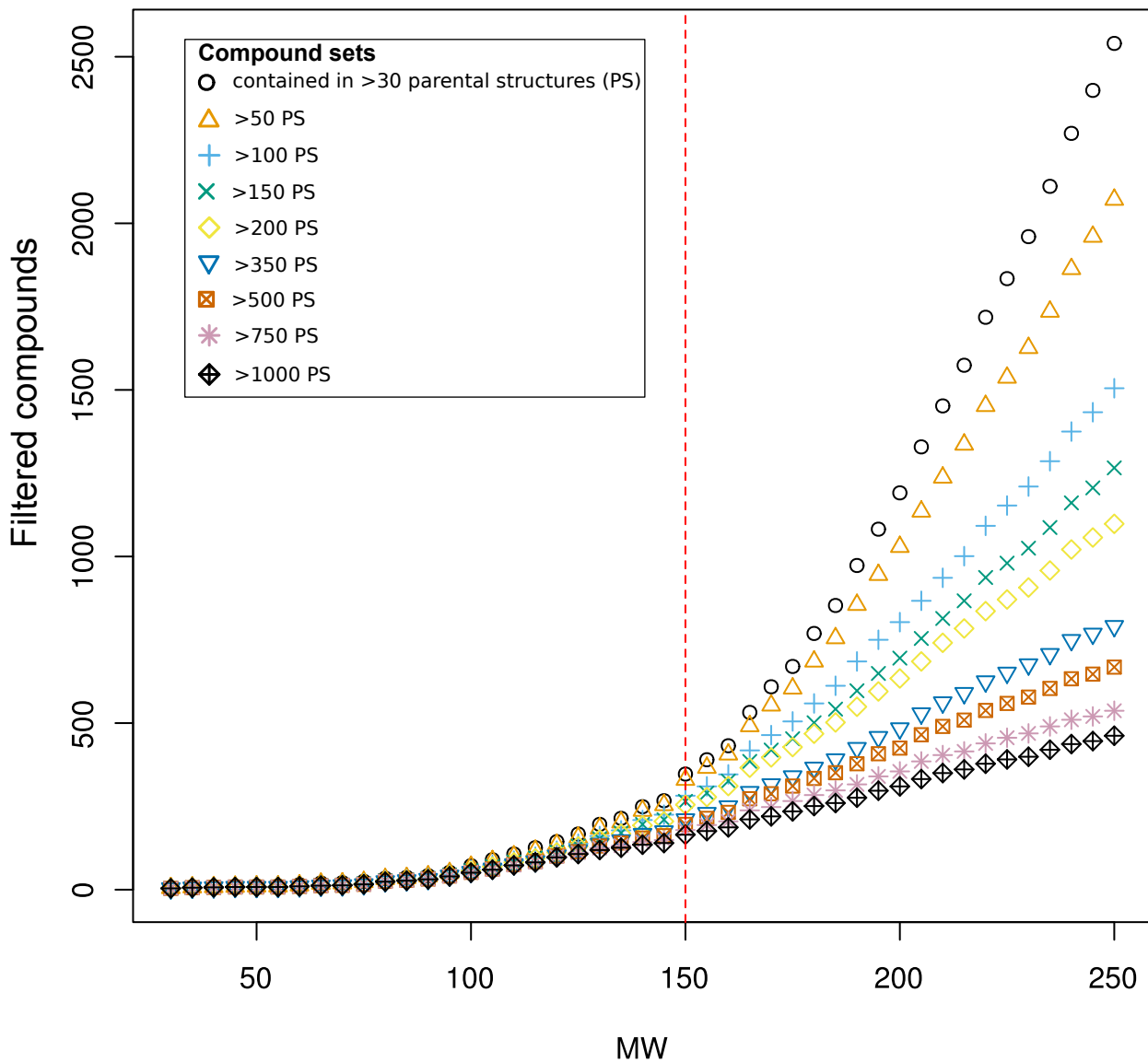

Supplement: S3 Fig — The plot shows the number of compounds involved in substructure similarity relationships that can be filtered out as a function of molecule size (MW) for different promiscuity threshold levels (different curves). PS = parental structures (those that contain a compound as part of its structure). (PDF) [file pntd.0004300.s003.pdf]

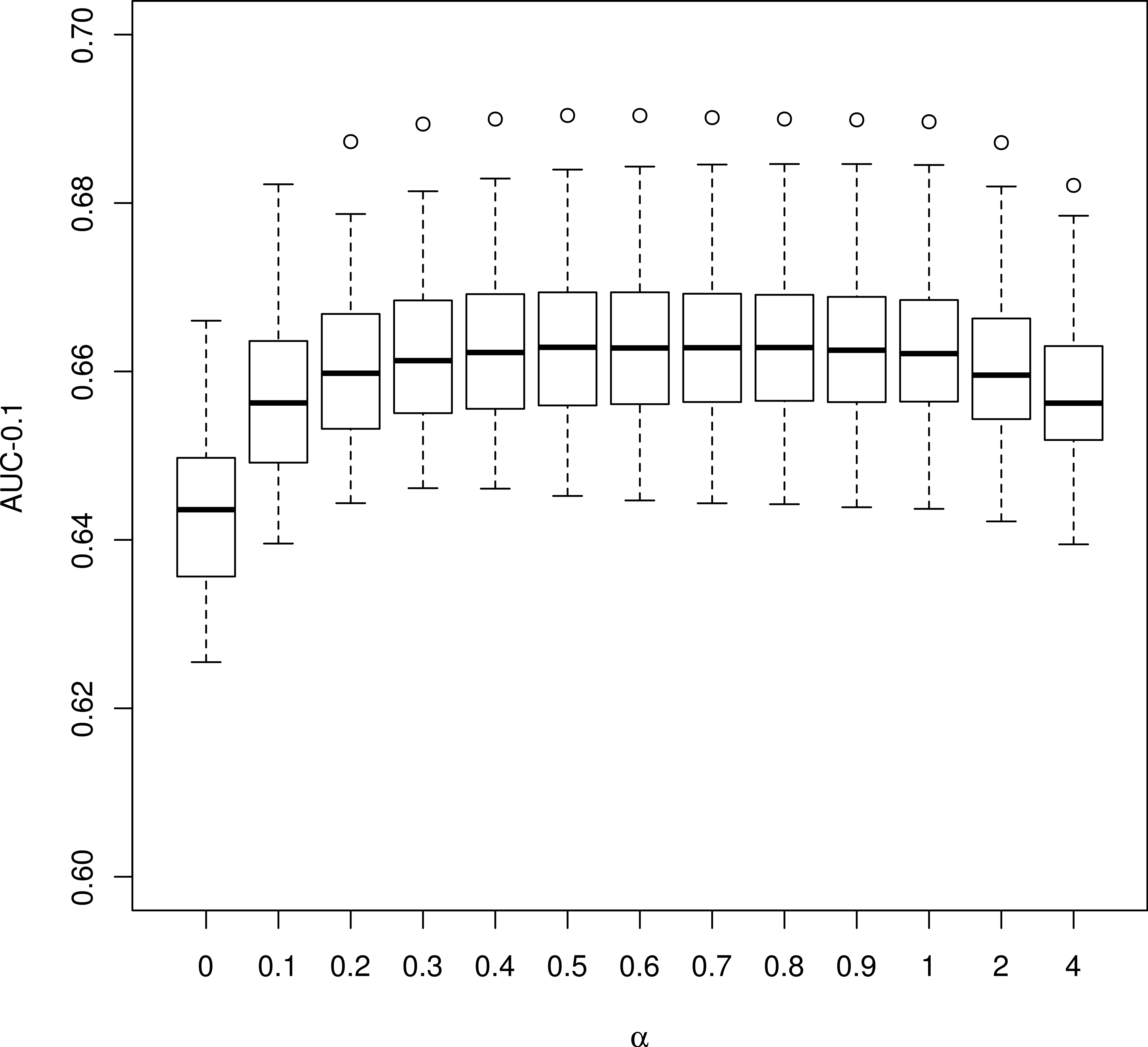

Supplement: S4 Fig — The figure shows the performance of 10-fold cross-validation target prioritization exercises in which all target-compound bioactivity links were removed for two query species (T. cruzi and M. musculus). Network projections were calculated using different values of the free parameter α. Overall we observed that differences in AUC-0.1 values were within 5% of tolerance. (TIFF) [file pntd.0004300.s004.tiff]
