# Supplementary material for: A Multilayer Network Approach for Guiding Drug Repositioning in Neglected Diseases
Source: PLoS Negl Trop Dis. 2016 Jan 6;10(1):e0004300. doi: 10.1371/journal.pntd.0004300 (PMC4703370; doi:10.1371/journal.pntd.0004300)
Supplement: S1 Table — We list the name of the organism and a brief summary of the taxonomic classification or grouping for each species. (PDF) [file pntd.0004300.s005.pdf]

Table S1. List of organisms with complete genomes included in our network model. We list the name of the organism and a brief summary of the taxonomic classification or grouping for each species.

Pathogens	
Plasmodium falciparum	Protozoa; Apicomplexa
Plasmodium berghei	
Plasmodium chabaudi	
Plasmodium knowlesi	
Plasmodium vivax	
Plasmodium yoelii	
Toxoplasma gondii	
Neospora caninum	
Babesia bovis	
Cryptosporidium hominis	
Cryptosporidium neoformans	
Cryptosporidium parvum	
Cryptosporidium muris	
Trypanosoma cruzi	Protozoa; Euglenozoa
Trypanosoma brucei	
Trypanosoma brucei gambiense	
Trypanosoma congolense	
Trypanosoma vivax	
Leishmania major	
Leishmania infantum	
Leishmania braziliensis	
Leishmania Mexicana	
Giardia lamblia	Protozoa; Metamonada
Giardia intestinalis	
Mycobacterium tuberculosis	Bacteria; Actinobacteria
Mycobacterium leprae	
Schistosoma mansoni	Metazoa; Trematoda (Helminths)
Brugia malayi	Metazoa; Nematoda (Helminths)
Wolbachia Brugia malayi	Bacteria; Alphaproteobacteria

Model Organisms	
Homo sapiens	Metazoa; Mammalia
Mus musculus	
Drosophila melanogaster	Metazoa; Arthropoda
Caenorhabditis elegans	Metazoa; Nematoda (Helminths)
Arabidopsis thaliana	Viridiplantae (green plants)
Oriza sativa	
Saccharomyces cerevisiae	Fungi; Ascomycota
Escherichia coli	Bacteria; Gammaproteobacteria
