# Supplementary material for: A Multilayer Network Approach for Guiding Drug Repositioning in Neglected Diseases
Source: PLoS Negl Trop Dis. 2016 Jan 6;10(1):e0004300. doi: 10.1371/journal.pntd.0004300 (PMC4703370; doi:10.1371/journal.pntd.0004300)
Supplement: S2 Table — Voting Scheme (VS) and Functional Flow (FF) network prioritization strategies were compared in terms of its AUC-0.1 performance (normalized scores were reported by using McClish Correction [56]). In spite of the more sophisticated procedure, FF performance did not improve the VS simpler performance. In the table we also show AUC-0.1 values corresponding to alternative versions of our affiliation network in which we removed one type of functional affiliation in each case. S = score used to weight EPF edges in the network (see main text); K = degree of each VP node; S/K = normalized score over the degree. In both cases, S/K network outperform performances. (PDF) [file pntd.0004300.s006.pdf]

S2 Table. Comparison between propose prioritization network methods. Voting Scheme (VS) and Functional Flow (FF) network prioritization strategies were compared in terms of its AUC-0.1 performance (normalized scores were reported by using McClish Correction [56]). In spite of the more sophisticated procedure, FF performance did not improve the VS simpler performance. In the table we also show AUC-0.1 values corresponding to alternative versions of our affiliation network in which we removed one type of functional affiliation in each case. S = score used to weight E_{PF} edges in the network (see main text); K = degree of each V_P node; S/K = normalized score over the degree. In both cases, S/K network outperform performances.

	Network Model			
	VS		FF	
	G_r	G_{rk}	G_r	G_{rk}
Mus musculus	0.64	**0.72**	0.65	**0.66**
Trypanosoma cruzi	0.52	**0.81**	0.52	**0.72**
