# Supplementary material for: A Multilayer Network Approach for Guiding Drug Repositioning in Neglected Diseases
Source: PLoS Negl Trop Dis. 2016 Jan 6;10(1):e0004300. doi: 10.1371/journal.pntd.0004300 (PMC4703370; doi:10.1371/journal.pntd.0004300)
Supplement: S1 Text — We analyzed in detail the discrepancies among G’rk and G’r based prioritizations. We showed how the performance discrepancy happened to be related to a strong correlation that existed between G’r -prioritization scores and network´s connectivity topological features. (PDF) [file pntd.0004300.s009.pdf]

Relevance scoring scheme and predictive power

As stated in the main text, prioritizations based on the G'_{rk} network model presented the best performance for the tested cases. On the other hand prioritizations considering G'_r resulted in much poorer performances, especially for the *T. cruzi* case, for which almost random classification performance was reported. Interestingly, the origin of the performance discrepancies between both network-based approaches happened to be related to a strong correlation that existed between G'_r -prioritization scores and the *strength,* s_i, a connectivity topological feature, of V_p nodes defined as:

$$s_i = \sum_{j \in \mathcal{N}(i)} w_{ij} \qquad [0]$$

where $\boldsymbol{\mathcal{N}}(\boldsymbol{i})$ is the set of neighbors of node-i and w_{ij} is the weight of the edge between node *i* and *j*.

Left panels in the Figure (see below) display the Spearman correlation level observed between a node's *strength* and the corresponding prioritization scores for the top *L*% best ranked proteins according to G'_{rk} (black triangles) and G'_r (red diamonds) network models. Panels (A) and (C) correspond to the *M. musculus* and *T. cruzi* cases respectively. A consistent high correlation level can be recognized between the first *L%* G'_r-prioritization scores and the node *strength* signal for a wide range of *L* values. Moreover slightly higher correlation levels are found when the very top ranked proteins are considered (i.e. $L \to 0$). Noticeably, these results suggest that the G'_r prioritization is *a priori* independent of any given seed set, as it is strongly influenced by topological features. Proteins annotated to promiscuous functional affiliation nodes (nodes with high degree) and/or affiliation nodes with high proportion of drug target annotated proteins (i.e. nodes connected through high relevance score in their E_{DP} edges) are expected *a priori* to appear as top-ranked candidates under this prioritization scheme.

A completely different scenario was observed for G'_{rk} prioritizations. In particular, for the top 1% scoring proteins (i.e. L=0.01, which corresponds to 134 proteins) only a minimal correlation of ρ~0.2 was observed for between *strength* and prioritization score for the analyzed organisms.

Panels (B) and (D) in the Figure display boxplots associated to the strength distribution of non-target and target proteins for the top 10% ranked proteins according to the G'_r network model corresponding to *M. musculus* and *T. cruzi* respectively. It can be seen that these target proteins do not display high strength levels as a general rule, what could explain the low AUC-01 values displayed by this network model.

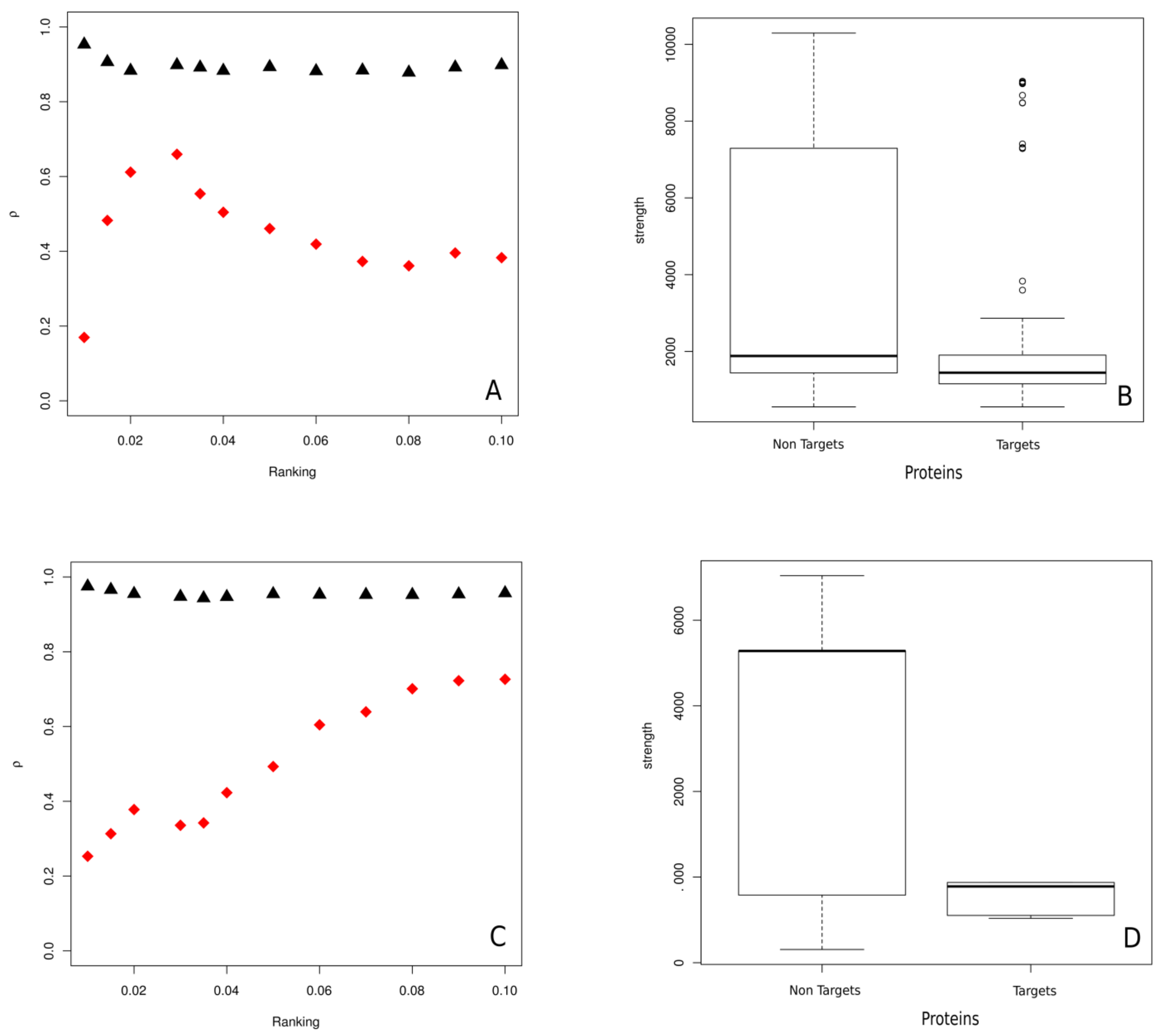

Figure: Spearman Rank Correlation Strength. Spearman correlation level observed between node *strengths* and the corresponding prioritization scores for the top *L*% best ranked proteins according to G'_{rk} (black triangles) and G'_{r} (red diamonds) network models are shown on left panels. Panels (A) and (C) correspond to the *M. musculus* and *T. cruzi* cases respectively. Panels (B) and (D) display boxplots associated to the strength distribution of non-target and target proteins for the top 10% ranked proteins according to the G'_{r} network model for *M. musculus* and *T. cruzi* respectively.

Spearman correlation level observed between node's *strengths* and the corresponding prioritization scores for the top *L*% best ranked proteins according to G'_{rk} (black triangles) and G'_{r} (red diamonds) network models. Panels (A) and (C) correspond to the *M. musculus* and *T. cruzi* cases respectively. Panels (B) and (D) in Supplementary Fig. 5 display boxplots associated to the strength distribution of non-target and target proteins for the top 10% ranked proteins according to the G'_{r} network model corresponding to *M. musculus* and *T. cruzi* respectively.
